# Systematic Analysis of Circadian Genes in a Population-Based Sample Reveals Association of *TIMELESS* with Depression and Sleep Disturbance

**DOI:** 10.1371/journal.pone.0009259

**Published:** 2010-02-18

**Authors:** Siddheshwar J. Utge, Pia Soronen, Anu Loukola, Erkki Kronholm, Hanna M. Ollila, Sami Pirkola, Tarja Porkka-Heiskanen, Timo Partonen, Tiina Paunio

**Affiliations:** 1 Public Health Genomics Unit, National Institute for Health and Welfare, Helsinki, Finland; 2 Department of Physiology, University of Helsinki, Helsinki, Finland; 3 Department of Psychiatry, Helsinki University Central Hospital, Helsinki, Finland; 4 Institute for Molecular Medicine Finland FIMM, University of Helsinki, Helsinki, Finland; 5 Department of Chronic Disease Prevention, Population Studies Unit, National Institute for Health and Welfare, Turku, Finland; 6 Department of Mental Health and Substance Abuse Services, National Institute for Health and Welfare, Helsinki, Finland; Leiden University Medical Center, Netherlands

## Abstract

Disturbances in the circadian pacemaker system are commonly found in individuals with depression and sleep-related problems. We hypothesized that some of the canonical circadian clock genes would be associated with depression accompanied by signs of disturbed sleep, early morning awakening, or daytime fatigue. We tested this hypothesis in a population-based sample of the Health 2000 dataset from Finland, including 384 depressed individuals and 1270 controls, all with detailed information on sleep and daytime vigilance, and analyzed this set of individuals with regard to 113 single-nucleotide polymorphisms of 18 genes of the circadian system. We found significant association between *TIMELESS* variants and depression with fatigue (D+FAT+) (rs7486220: pointwise P = 0.000099, OR = 1.66; corrected empirical P for the model of D+FAT+  = 0.0056; haplotype ‘*C-A-A-C*’ of rs2291739-rs2291738-rs7486220-rs1082214: P = 0.0000075, OR = 1.72) in females, and association to depression with early morning awakening (D+EMA+) (rs1082214: pointwise P = 0.0009, OR = 2.70; corrected empirical P = 0.0374 for the model D+EMA+; haplotype ‘*G-T*’ of rs7486220 and rs1082214: P = 0.0001, OR = 3.01) in males. There was significant interaction of gender and *TIMELESS* (for example with rs1082214, P = 0.000023 to D+EMA+ and P = 0.005 to D+FAT+). We obtained supported evidence for involvement of *TIMELESS* in sleeping problems in an independent set of control individuals with seasonal changes in mood, sleep duration, energy level and social activity in females (P = 0.036, ® = 0.123 for rs1082214) and with early morning awakening or fatigue in males (P = 0.038 and P = 0.0016, respectively, for rs1082214). There was also some evidence of interaction between *TIMELESS* and *PER1* in females to D+FAT+ as well as between *TIMELESS* and *ARNTL*, *RORA* or *NR1D1* in males to D+EMA+. These findings support a connection between circadian genes and gender-dependent depression and defective sleep regulation.

## Introduction

The circadian rhythm is an inherent cycle of approximately 24 hours entrained by environmental cues, particularly by light-dark transitions [1]. A region of the brain, the suprachiasmatic nucleus (SCN) of the anterior hypothalamus, operates as the master biological clock [2]. Light information goes from the eye to the SCN via the retino-hypothalamic pathway [3], [4], and the neurons within the SCN mediate a series of interlinked autoregulatory transcriptional/translational feedback loops [5], [6]. The key transcriptional activator of the molecular clock consists of a heterodimer between either the clock homolog protein (*CLOCK*, alias *bHLHe8*) or the neuronal PAS domain protein 2 (*NPAS2,* alias *bHLHe9*) and the aryl hydrocarbon receptor nuclear translocator-like protein (*ARNTL*, alias *BMAL1*) that binds to E-box elements in the promoter of three period (*PER*) and two cryptochrome (*CRY*) genes, thereby activating their transcription [7], [8]. A number of other genes, such as nuclear receptor subfamily 1, group D, member 1 (*NR1D1*), RAR-related orphan receptor A (*RORA*), and timeless homolog (Drosophila) (*TIMELESS*), are involved in the feedback loops. The central clock is a key regulator of many bodily functions that follow a circadian rhythm, such as sleep and wakefulness, thermoregulation, and glucose homeostasis and fat metabolism.

Functional regulation of sleep is essential for health, and sleep is associated with energy restoration [9]. Individuals who experience poor sleep regulation often suffer from fatigue, increased risk of accidents as well as poor performance and motivation [10], [11]. People with major depression commonly experience changes in sleep regulation that are seen as abnormal total sleep duration, poor sleep efficiency, overwhelming rapid-eye-movement sleep and early morning awakening [12], [13], [14]. A number of epidemiological studies have demonstrated that poor sleep often precedes the onset of depression [15], [16], [17], [18], [19], [20]. Utilizing a cohort of 18,631 same-sex twins, we recently showed that poor sleep quality correlates with subsequent onset of depressed mood, whereas the converse relation was not supported [21]. Poor sleep quality may reflect a relative shortage of slow-wave sleep, which has been considered as a predictor of recurrent depressive episodes [22].

The ever-increasing occupational and social demands of modern life may predispose individuals to instability in their circadian rhythm, which seems to be an intrinsic feature of mood disorders; moreover, circadian instability disrupts hormone and neurotransmitter release [23]. Furthermore, a disrupted circadian rhythm can affect the sleep-wake cycle [24], [25], which is one of the early symptoms of mania [26], and disruption of circadian activity is apparent in bipolar patients even when they are not acutely ill [27]. The circadian pacemaker generates the sleep-wake cycle [28], and it is the phase relationship between the sleep-wake cycle and the circadian pacemaker during entrainment that promotes the consolidation of sleep and wakefulness [29]. At night, the SCN receives specific feedback from sleep stages [30], and an excess of rapid-eye-movement sleep may result in the advanced phase position of the circadian rhythms in depressed individuals.

A number of studies have tested variants of genes that control the circadian system for their association with mood disorder [31], [32], [33], [34], [35], [36], [37], [38], [39]. However, many of the findings await replication in other samples and populations. Perhaps the most convincing evidence so far has emerged from bipolar disorder with some evidence of association with period homolog 3 (Drosophila) (*PER3*), *ARNTL* [36], basic helix-loop-helix family, member e40 (*BHLHE40*), casein kinase 1, epsilon (*CSNK1E*), and *CLOCK* [40]. Interestingly, *Npas2* deficient mice [41] as well as *Clock* mutant mice display a behavior profile similar to the manic state in bipolar disorder [42].

A role for circadian gene dysfunction has been established among the human sleep disorders, a subset of insomnias associated with circadian changes in the timing of sleep in humans [43], the most striking evidence for which is the familial advanced sleep-phase syndrome (ASPS) in which a phosphorylation site mutation of period homolog 2 (Drosophila) (*PER2*) was found to co-segregate with the disease in one extended family [44]. *PER2* has also been reported to be associated with morning preference [45]. There is some evidence for association of *PER3* [45], [46], [47], [48] and *CSNK1E* with delayed sleep phase syndrome (DSPS) [49]. In addition to the timing of sleep phase as evidenced with ASPS and DSPS, circadian clock genes may contribute to the duration of sleep phase, as demonstrated with basic helix-loop-helix family, member e41 (*BHLHE41*) whose amino acid changing mutation was found to co-segregate with the short sleep phenotype in a small nuclear family with two affected individuals [50]. Furthermore, differences in the sleep-wake structure, sleep propensity, and cognitive performance during sleep loss between individuals have been predicted by certain polymorphisms in *PER3* [51]. Intriguingly, the mutant mouse models of many clock genes, such as *Arntl*, *Clock*, *Npas2*, *Cry1* and *Cry2*, also have alterations in sleep duration and homeostasis [52], [53], [54], [55].

We have previously shown that there are gender-dependent and symptom-specific differences in the genetic background of depression at the population level. Some of the susceptibility genes, such as cAMP responsive element binding protein 1 (*CREB1*) in males, affect the core component of depressive disorder with depressive mood and anhedonia (unrelated to sleep disturbance), whereas others, such as tryptophan hydroxylase 2 (*TPH2*) or glutamate decarboxylase 1 (*GAD1*) in females, are more strongly associated with mood disorder only when accompanied with disturbed sleep [56]. We expanded this hypothesis to include genes from the circadian system and assumed that some of the circadian clock genes would be associated with depression accompanied by signs of disturbed sleep, early morning awakening, or daytime fatigue. We also hypothesized that seasonal fluctuation is common in number of the patients with mood disorder [57], and the same genes would associate with seasonal variations in mood and behavior.

We tested these hypotheses in a population-based sample of the Health 2000 dataset from Finland, comprising 384 depressed individuals and 1270 controls with detailed information on sleep, daytime vigilance and seasonality features, and analyzed this set of individuals with respect to 113 single-nucleotide variants from 18 genes of the circadian system.

## Results

A total of 113 single-nucleotide polymorphisms (SNPs) spanning 18 genes from the circadian system were genotyped in the Health 2000 population-based sample to investigate the genetic background of depression and its characteristic symptoms related to sleep disturbances. We first performed single-locus analyses of the variants that surviving Hardy-Weinberg equilibrium test (n = 106). These analyses were made separately for both genders with three models of depression. Tables 1 and 2 show pointwise permuted P-values from those analysis with indication if the P-value over the model in question was <0.05. This was followed by haplotype association analyses (Tables 3 and 4), and by interaction analyses (Table 5) on variants selected based on results from the single-locus analyses. Complete data from permutation-based allelic association analyses for depression and its subtypes, linear regression analysis for global seasonality score (GSS), and linkage disequilibrium (LD) patterns for all genotyped genes are available in Tables S1, S2, S3 and S4, respectively.

**Table 1:** Association between depressive disorder, global seasonality score (GSS), and SNPs of the circadian clock genes in females from the Finnish population.

Gene	SNPs^a^	Allele^b^	Single locus analysis permutation-based P-value and OR (95% CI^c^)						MAF d					Global Seasonality Score^e^	
									*(D+)*				*(D–)*		
			*D+ vs. D–*		*D+EMA+ vs. D–EMA–*		*D+FAT+ vs. D–FAT–*		*EMA– FAT–*	*EMA+ FAT+*	*EMA+ FAT–*	*EMA– FAT+*	*EMA– FAT–*	*GSSf1 P-value*	*GSSf2 P-value*
*TIMELESS*	rs2291739	T/C	0.072	1.21 (0.97–1.46)	0.060	1.31(0.98–1.74)	**0.0004**	1.51 (1.18–1.88)	0.3	0.47	0.3	0.44	0.36	0.453	0.486
*TIMELESS*	rs2291738	G/A	0.055	1.22 (0.98–1.48)	0.110	1.26 (0.96–1.71)	**0.0005**	1.52 (1.20–1.91)	0.37	0.46	0.33	0.47	0.43	0.204	0.289
*TIMELESS*	rs7486220	G/A	**0.009**	1.34 (1.07–1.63)	**0.003**	1.55 (1.16–2.08)	**0.000099** *	1.66 (1.31–2.10)	0.26	0.46	0.23	0.40	0.32	0.188	0.496
*TIMELESS*	**rs1082214**	C/T	**0.030**	0.60 (0.39–0.94)	**0.012**	0.33 (0.14–0.75)	0.126	0.66 (0.40–1.11)	0.03	0.02	0.03	0.06	0.07	0.829	**0.016**
*ARNTL*	rs1982350	A/G	**0.033**	1.24 (1.04–1.55)	0.547	1.09 (0.82–1.46)	**0.04**	1.27 (1.03–1.63)	0.48	0.47	0.46	0.44	0.45	0.395	0.820
*ARNTL*	rs6486121	C/T	**0.043**	0.80 (0.64–0.96)	0.159	0.81 (0.60–1.07)	**0.044**	0.78 (0.61–0.97)	0.46	0.44	0.43	0.40	0.48	0.631	0.842
*ARNTL*	rs969485	A/G	0.121	0.81 (0.63–1.05)	0.587	0.90 (0.63–1.28)	**0.026**	0.70 (0.53–0.95)	0.17	0.18	0.4	0.17	0.23	0.934	0.707
*RORA*	rs4774370	T/C	**0.045**	0.75 (0.57–0.98)	0.449	0.85 (0.57–1.20)	0.122	0.78 (0.57–1.03)	0.11	0.18	0.16	0.17	0.21	0.640	0.966
*RORA*	rs8027829	C/T	**0.048**	0.81 (0.66–1.01)	**0.026**	0.70 (0.51–0.96)	0.300	0.88 (0.69–1.13)	0.33	0.30	0.16	0.35	0.36	0.609	0.949
*RORA*	rs4774388	A/G	0.093	0.76 (0.58–1.09)	0.128	0.68 (0.45–1.13)	**0.010**	0.61 (0.44–0.94)	0.19	0.10	0.1	0.07	0.14	0.599	0.982
*NFIL3*	rs1619450	T/C	**0.022**	0.64 (0.46–0.95)	0.316	0.76 (0.48–1.29)	**0.017**	0.59 (0.39–0.91)	0.08	0.09	0.06	0.05	0.11	0.969	0.737
*NFIL3*	rs10991925	A/G	**0.033**	1.31 (1.01–1.65)	0.773	1.06 (0.71–1.47)	0.227	1.19 (0.87–1.54)	0.25	0.17	0.3	0.24	0.19	0.949	0.849
*CSNK1E*	rs135745	C/G	0.129	1.17 (0.95–1.42)	0.107	1.27 (0.93–1.66)	**0.015**	1.34 (1.05–1.67)	0.41	0.46	0.4	0.48	0.45	0.105	0.273
*CRY2*	rs10838524	A/G	**0.038**	1.24 (1.03–1.55)	**0.010**	1.45 (1.11–1.99)	0.104	1.22 (0.99–1.58)	0.48	0.45	0.3	0.49	0.46	0.667	0.624

a SNPs yielding permutated pointwise p-values<0.05 in the single-locus analyses.

b Allele: Major/Minor.

c 95% confidence intervals for the odds ratio (OR).

d Minor allele frequency in non-overlapping groups of cases *(D+)* and controls*(D–)* stratified by the absence or presence of early morning awakening or fatigue.

e The P-values for GSS factor 1 (metabolic factor) and factor 2 (mental factor) association were generated using the linear regression model. Bold face SNP signify *P*-values<0.05, β (Regression coefficient)  = −0.110.

*D+, patients with depression.*

*D–, controls (no depression).*

*D+EMA+, depressed patients with early morning awakening.*

*D–EMA–, controls without early morning awakening.*

*D+FAT+, depressed patients with fatigue.*

*D–FAT–, controls without fatigue.*

* Permutation-based corrected empirical P = 0.0056 over the model D+FAT+ vs. *D–FAT–* when all examined variants (n = 106) were considered.

**Table 2:** Association between depressive disorder, global seasonality score (GSS), and SNPs of the circadian clock genes in males from the Finnish population.

Gene	SNPs^a^	Allele^b^	Single locus analysis permutation-based P-value “and OR (95% CI^c^)^”^						MAF d				Global Seasonality Score^e^	
									*(D+)*			*(D–)*		
			*D+ vs. D–*		*D+EMA+ vs. D–EMA–*		*D+FAT+ vs. D–FAT–*		*EMA– FAT–*	*EMA+ FAT+*	*EMA– FAT+*	*EMA– FAT–*	*GSSf1 P-value*	*GSSf2 P-value*
*TIMELESS*	rs1082214	C/T	0.067	1.56 (1.01–2.58)	**0.0009** *	2.70 (1.59–4.64)	**0.037**	1.72 (1.09–3.06)	0.03	0.15	0.03	0.06	0.272	0.544
*ARNTL2*	rs4964060	G/A	0.138	0.79 (0.61–1.08)	0.143	0.73 (0.51–1.11)	**0.028**	0.68 (0.51–0.96)	0.4	0.36	0.34	0.43	0.375	0.190
*ARNTL2*	rs7304939	C/T	0.073	0.6 (0.34–1.05)	0.133	0.54 (0.25–1.23)	**0.023**	0.46 (0.24–0.92)	0.15	0.05	0.06	0.10	0.122	0.609
*ARNTL2*	rs12299407	T/C	0.184	0.74 (0.49–1.11)	0.107	0.60 (0.33–1.11)	**0.046**	0.60 (0.37–0.98)	0.3	0.09	0.15	0.16	0.184	0.469
*ARNTL2*	rs1037921	A/G	0.282	0.70 (0.39–1.27)	0.068	0.39 (0.14–1.12)	**0.047**	0.47 (0.22–1.00)	0.16	0.02	0.07	0.07	0.075	0.782
*ARNTL2*	rs2289709	C/T	0.142	0.68 (0.42–1.10)	**0.02**	0.37 (0.16–0.88)	**0.014**	0.47 (0.25–0.87)	0.26	0.04	0.10	0.11	0.099	0.951
*ARNTL*	rs2290036	T/C	**0.010**	1.70 (1.13–2.51)	**0.014**	1.88 (1.09–3.09)	**0.041**	1.57 (0.98–2.37)	0.2	0.16	0.12	0.09	0.825	0.173
*ARNTL*	rs3816358	C/A	0.341	1.21 (0.78–1.72)	**0.046**	1.66 (0.99–2.63)	0.366	1.22 (0.76–1.79)	0.13	0.19	0.09	0.12	0.810	0.850
*ARNTL*	rs969485	A/G	0.069	1.34 (0.96–1.81)	0.067	1.48 (0.98–2.25)	**0.025**	1.49 (1.06–2.10)	0.2	0.30	0.25	0.21	0.877	0.247
*NPAS2*	rs12712083	A/G	0.091	0.78 (0.58–1.03)	0.464	0.87 (0.60–1.29)	**0.045**	0.72 (0.54–1.01)	0.4	0.39	0.35	0.44	0.541	0.666
*TIPIN*	rs2063690	C/G	0.165	1.37 (0.89–2.2)	0.059	1.72 (0.97–3.03)	**0.037**	1.71 (1.09–2.83)	0.03	0.13	0.09	0.07	0.919	0.569
*RORA*	rs1568717	G/T	0.259	1.20 (0.83–1.60)	**0.026**	1.60 (1.04–2.39)	0.392	1.17 (0.79–1.61)	0.2	0.29	0.15	0.21	0.088	0.148
*RORA*	rs893287	C/T	**0.037**	0.72 (0.55–0.99)	**0.050**	0.65 (0.43–0.99)	**0.050**	0.72 (0.53–1.02)	0.33	0.30	0.34	0.39	0.146	0.271
*PER1*	**rs885747**	C/G	0.787	1.04 (0.77–1.33)	**0.040**	0.66 (0.45–0.96)	0.705	0.93 (0.70–1.28)	0.4	0.40	0.45	0.49	**0.022**	0.371

a SNPs yielding permutated pointwise p-values<0.05 in the single-locus analyses.

b Allele: Major/Minor.

c 95% confidence intervals for the odds ratio (OR).

d Minor allele frequency in non-overlapping groups of cases *(D+)* and controls*(D–)* stratified by the absence or presence of early morning awakening or fatigue.

e The P-values for GSS factor 1 (metabolic factor) and factor 2 (mental factor) were generated using the linear regression model. Bold face SNP signify *P*-values<0.05, β (Regression coefficient)  = 0.069.

*D+, patients with depression.*

*D–, controls (no depression)*.

*D+EMA+, depressed patients with early morning awakening.*

*D–EMA–, controls without early morning awakening.*

*D+FAT+, depressed patients with fatigue.*

*D–FAT–, controls without fatigue.*

* Permutation-based corrected empirical P = 0.0374 over the model D+EMA+ vs. *D–EMA–* when all examined variants (n = 106) were considered.

**Table 3:** Haplotype association analysis of SNPs of the genes having associations of P<0.05 in the *single-locus analysis* in females.

Gene	SNPs	Haplotype	Frequency in cases	Frequency in controls	OR	P-values	Phenotype
*TIMELESS*	rs2291739- rs2291738	C-A	0.46	0.36	1.51	0.0006	D+FAT+
	rs2291738- rs7486220	A-A	0.41	0.28	1.81	0.0000077	D+FAT+
	rs7486220- rs1082214	A-C	0.44	0.32	1.65	0.000021	D+FAT+
	rs2291739-rs2291738-rs7486220	C-A-A	0.41	0.29	0.97	0.00001	D+FAT+
	rs2291738-rs7486220-rs1082214	A-A-C	0.41	0.28	1.81	0.0000067	D+FAT+
	rs2291739-rs2291738-rs7486220-rs1082214	C-A-A-C	0.41	0.29	1.72	0.0000075	D+FAT+
*ARNTL*	rs3897902-rs969485	A-G	0.04	0.1	0.37	0.002	D+FAT+
*CRY2*	rs10838524-rs7123390	G-G	0.55	0.44	1.56	0.003	D+EMA+
*RORA*	rs4774370-rs1863270	C-T	0.08	0.12	0.61	0.002	D+

Odds ratio (OR).

*D+FAT+, depressed patients with fatigue.*

*D+EMA+, depressed patients with early morning awakening.*

*D+, patients with depression.*

**Table 4:** Haplotype association analysis of SNPs of the genes having associations of P<0.05 in the *single-locus analysis* in males.

Gene	SNPs	Haplotype	Frequency in cases	Frequency in controls	OR	P-values	Phenotype
*TIMELESS*	rs2291738- rs7486220	A-G	0.21	0.14	1.62	0.037	D+EMA+
	rs7486220- rs1082214	G-T	0.16	0.06	3.01	0.0001	D+EMA+
	rs2291739-rs2291738-rs7486220	C-A-G	0.16	0.08	2.20	0.006	D+EMA+
	rs2291738-rs7486220-rs1082214	A-G-T	0.11	0.05	2.36	0.004	D+EMA+
	rs2291739-rs2291738-rs7486220-rs1082214	C-A-G-T	0.12	0.05	2.61	0.003	D+EMA+
*ARNTL*	rs2290036-rs1868049	C-C	0.15	0.09	1.79	0.008	D+

Odds ratio (OR).

*D+EMA+, depressed patients with early morning awakening.*

*D+, patients with depression.*

**Table 5:** Interaction analysis of *TIMELESS* variants with all other genotyped circadian genes.

Chr1	SNP1	Gene1	Chr2	SNP2	Gene2	P-values	OR	Gender	Phenotype
12	rs2291739	*TIMELESS*	17	rs3027188	*PER1*	0.019	0.51	Females	D+FAT+
12	rs2291739	*TIMELESS*	17	rs2253820	*PER1*	0.046	0.59	Females	D+FAT+
12	rs2291739	*TIMELESS*	12	rs4964052	*ARNTL2*	0.049	0.71	Females	D+FAT+
12	rs2291739	*TIMELESS*	12	rs922270	*ARNTL2*	0.05	1.65	Females	D+FAT+
12	rs2291738	*TIMELESS*	2	rs1811399	*NPAS2*	0.022	1.59	Females	D+FAT+
12	rs2291738	*TIMELESS*	22	rs7289981	*CSNK1E*	0.042	0.57	Females	D+FAT+
12	rs7486220	*TIMELESS*	17	rs3027188	*PER1*	0.008	0.45	Females	D+FAT+
12	rs7486220	*TIMELESS*	17	rs2253820	*PER1*	0.044	0.57	Females	D+FAT+
12	rs1082214	*TIMELESS*	11	rs2290036	*ARNTL*	0.028	0.09	Females	D+FAT+
12	rs1082214	*TIMELESS*	11	rs4757151	*ARNTL*	0.037	0.44	Females	D+FAT+
12	rs1082214	*TIMELESS*	4	rs10462028	*CLOCK*	0.031	2.19	Females	D+FAT+
12	rs2291739	*TIMELESS*	15	rs2290430	*RORA*	0.005	10.1	Males	D+EMA+
12	rs2291739	*TIMELESS*	11	rs1868049	*ARNTL*	0.0006	4.36	Males	D+EMA+
12	rs2291739	*TIMELESS*	11	rs4757151	*ARNTL*	0.002	2.31	Males	D+EMA+
12	rs2291739	*TIMELESS*	11	rs3897902	*ARNTL*	0.003	4.18	Males	D+EMA+
12	rs2291739	*TIMELESS*	11	rs3816358	*ARNTL*	0.019	0.40	Males	D+EMA+
12	rs2291739	*TIMELESS*	11	rs969485	*ARNTL*	0.043	2.08	Males	D+EMA+
12	rs2291739	*TIMELESS*	12	rs922270	*ARNTL2*	0.027	0.21	Males	D+EMA+
12	rs2291738	*TIMELESS*	11	rs4757151	*ARNTL*	0.004	2.23	Males	D+EMA+
12	rs2291738	*TIMELESS*	11	rs1868049	*ARNTL*	0.005	3.16	Males	D+EMA+
12	rs2291738	*TIMELESS*	11	rs3897902	*ARNTL*	0.006	3.76	Males	D+EMA+
12	rs2291738	*TIMELESS*	11	rs3816358	*ARNTL*	0.041	0.46	Males	D+EMA+
12	rs2291738	*TIMELESS*	17	rs2289591	*PER1*	0.010	2.72	Males	D+EMA+
12	rs2291738	*TIMELESS*	15	rs2290430	*RORA*	0.017	6.53	Males	D+EMA+
12	rs2291738	*TIMELESS*	19	rs3745733	*DBP*	0.031	2.25	Males	D+EMA+
12	rs2291738	*TIMELESS*	1	rs3753503	*PER3*	0.039	8.42	Males	D+EMA+
12	rs2291738	*TIMELESS*	12	rs3809237	*CRY1*	0.045	1.75	Males	D+EMA+
12	rs7486220	*TIMELESS*	15	rs4774370	*RORA*	0.003	3.12	Males	D+EMA+
12	rs7486220	*TIMELESS*	1	rs3753503	*PER3*	0.031	5.18	Males	D+EMA+
12	rs7486220	*TIMELESS*	15	rs16943429	*RORA*	0.038	2.16	Males	D+EMA+
12	rs1082214	*TIMELESS*	17	rs2269457	*NR1D1*	0.003	3.97	Males	D+EMA+
12	rs1082214	*TIMELESS*	15	rs2028122	*RORA*	0.006	0.20	Males	D+EMA+
12	rs1082214	*TIMELESS*	15	rs3759785	*TIPIN*	0.020	11.56	Males	D+EMA+
12	rs1082214	*TIMELESS*	15	rs2063690	*TIPIN*	0.041	3.80	Males	D+EMA+

Chr: Chromosomes.

P-values and Odds ratios (OR) were generated using the logistic regression model. None of the P-values remained significant (P<0.05) when considering the number of tests performed (408 in both genders).

*D+FAT+, depressed patients with fatigue.*

*D+EMA+, depressed patients with early morning awakening.*

### Association to Depression in Females

#### Single-locus analyses in cases and controls

Single-locus analysis of females (n = 967) suggested an association (P<0.05) between depression and depression accompanied by signs of disturbed sleep with 14 SNPs from six circadian-related genes: *TIMELESS*, *ARNTL*, *RORA*, nuclear factor, interleukin 3 regulated (*NFIL3*), *CSNK1E*, and *CRY2* (Table 1; full data available in Table S1). The statistically strongest evidence was for the association of *TIMELESS* rs7486220 with depression and fatigue (pointwise P = 0.000099, odds ratio (OR)  = 1.66). This was the only variant that survived correction for multiple testing in females (corrected empirical P for the model D+FAT+  = 0.0056, Bonferroni corrected P for all models of the study  = 0.033). The associated minor allele ‘*A*’ of rs7486220 occurred more frequently in cases with excessive daytime fatigue (f = 0.40 in cases with fatigue, f = 0.32 in healthy controls). In addition, three *ARNTL* variants had modest association with depression alone or depression with fatigue, with strongest evidence being for rs969485 associated with depression and fatigue (P = 0.026, OR = 0.70). Three *RORA* variants also showed modest evidence for association with depression alone or with fatigue, with the best evidence for an intronic region SNP, rs4774388, that was associated with depression and fatigue (P = 0.01, OR = 0.61). Two *NFIL3* variants were modestly associated with depression and fatigue, with best evidence for rs1619450 (P = 0.017, OR = 0.59); there was a similar association for one *CSNK1E* variant, rs135745 (P = 0.015, OR = 1.34). Finally, a *CRY2* intronic variant, rs10838524, was associated weakly with depression and early morning awakening (P = 0.010, OR = 1.45).

*Haplotype analysis.* We then tested for an association of haplotypes comprising those variants that had shown evidence for association in the single-locus analyses (P<0.05, see Table 1) and their adjacent variants (Table 3). Those analyses provided further evidence for *TIMELESS*. The two-SNP haplotype ‘*A-A*’ of rs2291738 and rs7486220 occurred significantly more frequently in cases with fatigue (f = 0.41 in cases, f = 0.28 in controls; P = 0.0000077, OR = 1.81) as did haplotype ‘*A-C*’ of rs7486220 and rs1082214 (f = 0.44 in cases, f = 0.32 in controls, P = 0.000021, OR = 1.65). Owing to allelic coherence of these findings, we also tested for three-SNP and four-SNP haplotypes of *TIMELESS* and found significant overall association of the haplotype ‘*C-A-A-C*’ of rs2291739-rs2291738-rs7486220-rs1082214 (f = 0.41 in cases, f = 0.29 in controls; P = 0.0000075, OR = 1.72). The pairwise LD between these four markers was relatively high (D' =  0.99, 0.83, and 0.93, respectively).

The allelic haplotype ‘*A-G’* of SNPs rs3897902 and rs969485 in *ARNTL* also confirmed the suggestive association observed in the single-SNP analysis with depression and fatigue (P = 0.002, OR = 0.37, f = 0.04 in cases, f = 0.1 in controls; D' = 0.97), as did the haplotype ‘*G-G*’ of rs10838524 and rs7123390 in *CRY2* with depression and early morning awakening (P = 0.003, OR = 1.56, f = 0.55 in cases, f = 0.44 in controls; D' = 0.88) and haplotype ‘*C-T*’ of rs4774370 and rs1863270 in *RORA* with depression (P = 0.002, OR = 0.61, f = 0.08 in cases, f = 0.12 in controls, D' = 0.16).

#### Association analysis for GSS

The linear regression model in the complete sample of females and controlled for age and status for the GSS metabolic factor 1 (GSSf1) and GSS mental factor 2 (GSSf2) indicated modest associations for three variants: *TIMELESS* rs1082214 with GSSf2 (P = 0.016, ® = –0.110), *NFIL3* rs813498 with GSSf1 (P = 0.008, ® = –0.261), and TIMELESS interacting protein (*TIPIN*) rs2063690 with GSSf2 (P = 0.005, ® = –0.121) in depressed females (Table 1 and Table S3).

### Association to Depression in Males

#### Single-locus analyses in cases and controls

Analysis of single SNPs in males (n = 687) yielded suggestive evidence for association (P<0.05) between depression and depression accompanied by signs of disturbed sleep with 14 SNPs from six genes: *ARNTL*, aryl hydrocarbon receptor nuclear translocator-like 2 (*ARNTL2*), *RORA*, *NPAS2*, *TIPIN* and period homolog 1 (Drosophila) (*PER1*) (Table 2; full data available in Table S2). The strongest evidence was for a relatively rare variant rs1082214 of *TIMELESS*, that associated with depression accompanied by early morning awakening with the minor allele ‘*T*’ (pointwise P = 0.0009, OR = 2.7, f = 0.15 in cases with early morning awakening, f = 0.03 in cases without early morning awakening, f = 0.06 in controls; corrected empirical P for the model D+EMA+ P = 0.0374, Bonferroni corrected P for all models of the study  = 0.22).

Altogether four *ARNTL2* variants were associated suggestively with depression accompanied by fatigue, with the best evidence being for rs7304939 (P = 0.023, OR = 0.46), and one variant rs2289709 with depression accompanied by early morning awakening (P = 0.02, OR = 0.37) and fatigue (P = 0.014, OR = 0.47). Three *ARNTL* variants showed moderate association with depression alone or with fatigue, with best evidence for rs2290036 and depression (P = 0.010, OR = 1.70). *NPAS2* rs12712083, as well as a non-synonymous coding SNP located on exon 5 rs2063690 of *TIPIN* (Ala_111_Gly), were associated with depression and fatigue (P = 0.045, OR = 0.72 and P = 0.037, OR = 1.71, respectively). Finally, *RORA* rs1568717 and *PER1* rs885747 showed suggestive evidence for association with depression and early morning awakening (P = 0.026, OR = 1.60, and P = 0.040, OR = 0.66, respectively).

#### Haplotype analysis

The two-SNP haplotype analyses provided further evidence for *TIMELESS* with evidence for association of a relatively rare haplotype ‘*G-T*’ of rs7486220 and rs1082214 with depression and early morning awakening (P = 0.0001; OR = 3.01, f = 0.16 in cases, f = 0.06 in controls; D’ = 0.93). The haplotype ‘*C-C*’ of rs2290036 and rs1868049 in *ARNTL* were associated with depression (P = 0.008, OR = 1.79, f = 0.15 in cases, f = 0.09 in controls; D' = 1.0) (Table 4).

#### Association analysis for GSS

The linear regression model for the GSS factors in the complete sample of males indicated weak association of *PER1* rs885747 with GSSf1 (P = 0.022, ® = 0.069) and of *CRY 1* rs2287162 with GSSf2 (P = 0.0009, ® = 0.082) (Table 2 and Table S3).

### Interaction Analyses

As circadian genes function coordinately in the molecular clock, we looked for interactions of other genotyped variants of circadian genes with *TIMELESS* variants that gave the strongest evidence for association in both genders. In females with depression accompanied by fatigue (D+FAT+), we found some evidence of interaction between *TIMELESS* rs7486220 and *PER1* rs3027188 (P = 0.008, OR = 0.45) (Table 5). In males with depression accompanied by early morning awakening (D+EMA+), there was also some evidence of interaction between *TIMELESS* rs2291739 and *ARNTL* rs1868049 (P = 0.0006, OR = 4.36), between *TIMELESS* rs7486220 and *RORA* rs4774370 (P = 0.003, OR = 3.12), and between *TIMELESS* rs1082214 and *NR1D1* rs2269457 (P = 0.003, OR = 3.97) (Table 5). However, none of the P-values remained significant (P<0.05) when considering the number of tests performed (4×102 = 408 in both genders).

In addition, we looked for interaction of gender with variants from *TIMELESS* for depression with sleep disturbances. There was strong evidence for interaction of rs7486220 or rs1082214 to depression with early morning awakenings (P = 0.0015 and 0.000023, respectively), as well as for depression with fatigue (P = 0.005 for both variants).

### Analyses of *TIMELESS* in the Second Sample Set

To further elucidate the role of *TIMELESS* in regulation of mood and sleep disturbances, we analyzed the gene in another set of individuals from the Health 2000 cohort. That sub sample was initially collected for genome wide association study on metabolic syndrome. We excluded all those individuals that had a diagnosis of depression and were thus already included in the original study samples. These “depression-free” samples comprised then 759 females and 753 males.

Analysis with rs1082214, the only variant from *TIMELESS* available in that dataset, revealed evidence for association to GSSf2 in females (P = 0.036, ® = 0.123) (Table 6), so that the allele ‘*C*’ that was related to depression in females was also related to higher level of seasonality changes in mood. Rs1082214 also associated with early morning awakening (P = 0.038, OR = 1.52, f = 0.09) or fatigue (P = 0.0016, OR = 1.79 f = 0.10) in males so that the allele ‘*T*” that was related to depression and sleep disturbances in males was also related to early morning awakenings and fatigue in this second set of individuals.

**Table 6:** Results for *TIMELESS* SNP rs1082214 in the second sample set.

Gene	SNP	Allele^a^	Single locus analysis permutation-based P-value “and OR (95% CI^b^)”				Global Seasonality Score^c^		Gender
			*D–EMA+ vs. D–EMA–*		*D–FAT+ vs. D–FAT–*		*GSSf1*	*GSSf2*	
*TIMELESS*	rs1082214	C/T	0.462	0.85 (0.56–1.28)	0.518	1.12 (0.80–1.57)	0.974	0.036*	Female
			0.038	1.52 (1.01–2.28)	0.0016	1.79 (1.25–2.57)	0.994	0.107	Male

a Allele: Major/Minor.

b 95% confidence intervals for the odds ratio (OR).

c The *P*-values for quantitative traits were generated using the linear regression model.

* β (Regression coefficient)  = 0.123.

*D-EMA+, controls with early morning awakening.*

*D–EMA–, controls without early morning awakening.*

*D-FAT+, controls with fatigue.*

*D–FAT–, controls without fatigue.*

## Discussion

Here, we report evidence that genes from the circadian system have a role in the induction of depression and its subtypes associated with presence of early morning awakening and fatigue. We found significant association of a common allelic variant of *TIMELESS* and depression with fatigue as well as seasonal variations in mood, sleep duration, energy level and social activity in females. We also found suggestive association for another rare variant of *TIMELESS* with depression and early morning awakening in males, and some evidence for interaction between *TIMELESS* and other circadian genes in depression and related sleep problems. These findings support a connection between circadian genes and gender-dependent depression and defective sleep regulation.

The biological function of TIMELESS is essential for resetting the biological clock. It interacts directly with the PER proteins, and it negatively regulates the ARNTL-CLOCK and ARNTL-NPAS2 complexes that induce the transactivation of *PER1* [58]. TIMELESS is also involved in DNA damage checkpoint responses. It interacts with CRY2 and with the cell cycle checkpoint protein CHK1 and the ataxia-telangiectasia mutated (ATM)–Rad3-related kinase–ATR-interacting protein (ATR–ATRIP) complex [59], and it may be specifically required for the ATR-CHK1 pathway in the replication checkpoint induced by ultraviolet light in the skin and retina. Of the four *TIMELESS* variants examined here, a SNP located in the 3' untranslated region (rs7486220) showed the strongest association with the minor allele ‘*A*’, increasing the risk for depression with fatigue 1.66-fold in females. This was statistically the strongest finding of the study (pointwise P = 0.000099) which was significant over the model D+FAT+ (permutation-based corrected empirical P = 0.0056) as well as over all models of the study (Bonferroni corrected P = 0.034). However, LD between all genotyped markers of *TIMELESS* was high (D' = 0.83–0.99), and overall the strongest evidence for association was obtained with the haplotype ‘*C-A-A-C*’ of rs2291739-rs2291738-rs7486220-rs1082214 spanning the entire gene in females (P = 0.0000075, OR = 1.72). In males, the minor allele ‘*T*’ of rs1082214 in the promoter region, overlapping an intronic region in the gene encoding major intrinsic protein of lens fiber (*MIP*), was associated with depression and early morning awakening (pointwise P = 0.0009, OR = 2.7; permutation-based corrected empirical P for the model D+EMA+  = 0.0374, Bonferroni corrected P for all models of the study  = 0.22), as was the haplotype ‘*G-T*’ of rs7486220 and rs1082214 (P = 0.0001, OR = 3.01).

Thus, we obtained strong evidence of a role for *TIMELESS* in the genetic background of depression with signs of sleep disturbance in both genders, but the associated alleles were not the same. Moreover, evidence for females was obtained for a wider chromosomal segment than that for males, for which only one single variant yielded statistically significant evidence for association (P<0.001). A chicken ovalbumin upstream promoter transcription factor (COUP-TF) binding site is located in the promoter region of *TIMELESS*. The target binding sequences for COUP-TFs are typically highly conserved and reportedly are involved in the repression of gene expression [60] although the natural ligand in humans is not known. COUP-TFs are classified as members of the steroid receptor family [61], and one study has shown that COUP-TFI plays an important role in mitigating estrogen-responsive gene expression [62]. This molecular mechanism may account for the *TIMELESS* expression and our finding of different susceptibility alleles in males and females.

To further elucidate the role of *TIMELESS* in the interplay of depression and sleep disturbances, we examined the variant rs1082214 in an independent set of control individuals from Health 2000. In females, we observed that the same allelic variant (allele ‘*C*’ of rs1082214) that was part of the high-risk haplotype for depression and fatigue also associated to higher level of seasonality changes in mood. The other allelic form of that SNP (minor allele ‘*T*’ of rs1082214) that had increased risk for depression with early morning awakenings and fatigue, also associated with early morning awakening (P = 0.038) or fatigue (P = 0.0016) in males of the second study sample. These findings suggest that in females, association of *TIMELESS* is specific to fatigue accompanying depression (rather than to the symptoms of fatigue alone) and to seasonal fluctuation of mood. The finding of an association between *TIMELESS* and symptoms of disturbed sleep *without* diagnosis of depressive disorder in males is highly intriguing and may constitute evidence for a sub clinical form of depression not revealed in the CIDI interview but manifested mainly by symptoms of disturbed sleep. Possible phenotypic differences may offer a challenge for further studies and the development of diagnostic classification.

Earlier studies have also revealed a role for *TIMELESS* in insomnia, mania [40], bipolar disorder type 1, schizophrenia, and schizoaffective disorder [37]. The allele ‘*G*’ of intronic SNP rs2291738 was associated with female depression in our study (Table 1), and the same allele also has been found to be associated with bipolar disorder type 1 [37]. This is particularly interesting as bipolar disorder and depression share clinical features, such as depressive episodes and cyclic recurrence of phases. According to family and twin studies, they share also at least part of their genetic background [63], [64], [65] and in longitudinal studies, there is a shift from depression to bipolar disorder [66]. On this perspective it is highly interesting that we also found an association between *TIMELESS* and seasonal variations in mood, sleep duration, energy level and social activity, representing features that are common to both unipolar and bipolar mood disorders [67].

We found suggestive evidence for genetic interaction between *TIMELESS* and a number of clock genes within the circadian pathways. These observations imply that genetic networks that control the circadian system are intimately involved in the susceptibility to depression and sleep-related problems. We note, however, that the statistical relevance for these findings was relatively modest.

Several *ARNTL* variants showed modest association with depression accompanied by fatigue in females. Out of them rs1982350 and rs6486121 are in relatively high LD with variants that have been related to susceptibility to hypertension and type 2 diabetes [68]; rs1982350 has also been associated with schizophrenia/schizoaffective disorder and bipolar 1 disorder [37]. Rs969485, one of the markers of a haplotype previously associated with hypertension [68], was here associated with depression and fatigue in both genders with different alleles. Consequently, one might hypothesize that altered levels or function of *ARNTL* may contribute to hypertension and type 2 diabetes via mechanisms related to disturbed sleep and mood. In addition, we obtained evidence for association of the promoter and intron 1 region of *RORA* with depression and sleep disturbances, and this same region has been earlier associated with severe obesity [69]. Thus, these findings may offer one possible molecular mechanism for the association between metabolic syndrome and depressive disorders in general population [70], [71].

Previous studies have demonstrated important associations of clock genes with sleep and mood disorders, as for example between *PER2* variants and familial ASPS [44], [72], between *PER3* variants and diurnal preference and DSPS [46], [47], and between *CLOCK* and mood disorders [32], [33], [34] and human diurnal preference [73]. None of the variants from these genes showed evidence for association with depression or its subtypes in the present study, although the SNPs examined in *PER3* here are either identical or tag those genotyped in the other studies (Figure S1). It is, however, noteworthy that our sample was small and underpowered to detect genetic risks <1.5 in females and <1.8 in males (see materials and methods). This is also the major limitation of the current study. On the other hand, by analyzing separately cases with sleep disturbances, we aimed at diminish genetic heterogeneity of our sample that was originally derived by careful ascertainment from the relatively homogeneous population of Finland, as reflected in genetic terms by extended LD patterns as compared to other populations [74]. Another clear limitation is the problem of multiple testing that may lead to spurious P-values and it is noteworthy that out of the all variants examined here, only associations with the *TIMELESS* variants rs7486220 in females and rs1082214 in males sustain after correction for the multiple testing. Ultimately, our findings need to be replicated in sufficiently powered cohorts of patients with information on both mood and sleep.

In conclusion, we present here a systematic report on polymorphisms in multiple circadian genes and their associations with depression and disturbed sleep. Our data support the involvement of circadian clock genes in the gender-specific regulation of mood and sleep. This finding may have clinical relevance considering that the prevalence of depressive disorder varies between males and females and further helps us understand the genetics of the circadian system and to develop strategies to address its dysfunction.

## Materials and Methods

### Ethics Statement

This study was conducted according to the principles expressed in the Declaration of Helsinki. The study was approved by the Institutional Review Board of Helsinki and Uusimaa Hospital District. All participants provided written informed consent for the collection of samples and subsequent analysis.

### First Sample Set

The study sample was recruited from the population-based Health 2000 program in Finland. The sampling design, target population, and methods of the survey have been reported elsewhere (http://www.ktl.fi/terveys2000/index.uk.html). The health status of all study subjects was evaluated by an interview conducted at home and a health examination monitored by physicians and trained nurses at the local healthcare center. These interviews included questions related to the quality of sleep as well as general and mental health problems.

The diagnosis of major depressive disorder or dysthymia involves the research version of the CIDI based on criteria from the Diagnostic and Statistical Manual of Mental Disorders (DSM-IV) for psychiatric disorders during the last 12 months [75]. The details of the predisposition for early morning awakening and fatigue have been published [56].

The study sample consisted of 1654 subjects, the youngest of which was 30 years of age. Of these, 384 were cases (259 females, mean age 49 years, and 125 males, mean age 48 years) (group D+). There were 1270 individuals in the control group (708 females, mean age 46 years, and 562 males, mean age 45 years) with no depression or any other psychiatric disorder according to the CIDI interview (group D–). The control group were matched by their age and gender to the cases (n = 384) and it comprised 392 additional individuals from the general population that did not have any sleep related problems or disorders (Table 7). Altogether, the sample was representative of the Finnish population (Figure S2).

**Table 7:** Features of the samples used.

Group	N (Females)	Age (Average ± SD)	N (Males)	Age (Average ± SD)
**First sample set**				
*patients with depression (D+)*	259	49.02±13.65	125	47.94±10.75
*depressed patients with early morning awakening (D+EMA+)*	109^a^	51.64±13.19	61^b^	48.85±9.85
*depressed patients with fatigue (D+FAT+)*	194^a^	50.10±14.06	103^b^	48.39±11.07
*controls (no depression) (D–)*	708	46.35±11.80	562	44.80±10.57
*controls without early morning awakening (D–EMA–)*	705	46.32±11.80	561	44.83±10.57
*controls without fatigue* (*D–FAT–)*	580	46.10±11.68	482	45.28±10.69
**Second sample set**				
*controls with early morning awakening (D–EMA+)*	274^c^	56.87±10.38	248^d^	54.29±10.30
*controls with fatigue (D–FAT+)*	388^c^	56.24±10.22	342^d^	48.56±11.42
*controls without early morning awakening (D–EMA–)*	412	69.05±7.77	419	48.82±11.84
*controls without fatigue (D–FAT–)*	349	56.97±10.48	383	53.08±10.64

Overlap in group’s D+EMA+ and D+FAT+ was ^a^94 for females and ^b^ 58 for males [56].

Overlap in the second sample set with *D–*EMA+ and *D–*FAT+ was ^c^165 for females and ^d^136 for males.

Seasonal variations in mood and behavior were investigated in the study sample with a two-factor solution for GSS on the Seasonal Pattern Assessment Questionnaire [76]. Factor 1 was considered as a metabolic factor (GSSf1) (weight and appetite) and factor 2 as a mental factor (GSSf2) (sleep duration, social activity, mood and energy level) for the season-bound variations that may feature depressive episodes [77].

### Second Sample Set

We analyzed an independent set of individuals from the Health 2000 survey in a sample that was initially selected for the study on metabolic disorder. We excluded all cases with a CIDI-based diagnosis of depression from that sample which then comprised 753 males (men age 47 years) and 759 females (mean age 53 years). Of them, 691 males and 695 females had information on GSS. There were 248 males who reported to have early morning awakenings and 342 had fatigue. The corresponding numbers in females were 274 and 388, respectively (Table 7).

### Genotyping Methods

Genomic DNA was isolated from peripheral blood leukocytes using a standard EDTA extraction procedure [78]. The circadian genes we chose were based on the literature (see Table 8). SNPs within these genes were selected using the International HapMap database (www.hapmap.org) (see Table S5). CEPH (Centre d'Etude du Polymorphisme Humain) genotype data of the International HapMap Project were referred to in order to cover the haplotype tagging SNPs (tagSNPs)**,** International HapMap Consortium [79]. We implemented the pairwise tagging method with an r^2^ threshold of 0.8 and minor allele frequency (MAF) of 0.1. For large genes (tagging SNP number >50) such as *NPAS2* and *RORA*, tagSNPs were selected evenly spaced throughout the gene. The flanking regions of the DNA sequences were derived from SNPper [80]. The extension primers for polymerase chain reaction were designed with MassARRAY Assay Design 3.1 software (Sequenom Inc., San Diego, CA, USA).

**Table 8:** List of studied circadian candidate genes.

Gene Symbol	Gene Name	References
*PER3*	period homolog 3 (Drosophila)	[36], [37], [46], [47], [48], [51]
*PER2*	period homolog 2 (Drosophila)	[37], [44], [45], [84]
*NPAS2*	neuronal PAS domain protein 2	[31], [84]
*CLOCK*	clock homolog (mouse)	[32], [33], [34], [35], [37], [40], [42], [73]
*NFIL3*	nuclear factor, interleukin 3 regulated	[85]
*BHLHE40*	basic helix-loop-helix family, member e40	[40], [86]
*CRY2*	cryptochrome 2 (photolyase-like)	[37], [54]
*ARNTL*	aryl hydrocarbon receptor nuclear translocator-like	[36], [37], [53], [68], [84]
*ARNTL2*	aryl hydrocarbon receptor nuclear translocator-like 2	[40]
*BHLHE41*	basic helix-loop-helix family, member e41	[50], [86]
*TIMELESS*	timeless homolog (Drosophila)	[37]
*CRY1*	cryptochrome 1 (photolyase-like)	[37], [54]
*RORA*	RAR-related orphan receptor A	[69]
*TIPIN*	TIMELESS interacting protein	[87]
*NR1D1*	nuclear receptor subfamily 1, group D, member 1	[40], [88], [89]
*PER1*	period homolog 1 (Drosophila)	[37]
*DBP*	D site of albumin promoter (albumin D-box) binding protein	[40]
*CSNK1E*	casein kinase 1, epsilon	[40], [49]

SNP genotyping was performed using MassARRAY iPLEX Gold platform (Sequenom Inc.) following the manufacturer's guidelines in 24- to 34-plex reactions in 384-well plates using a total reaction volume of 5 µl including 12.5 ng of genomic DNA. The qualities of genotypes were analyzed using MassARRAY Typer 4.0 software (Sequenom Inc.) and verified manually. As quality controls, eight duplicated DNA samples and eight water controls were included in each plate. The overall average genotyping success rate for the SNP data was ≥95%, and MAF was ≥5%.

Hardy-Weinberg equilibrium was monitored using Haploview version 4.1 [81], and a cutoff of p<0.05 was applied. Seven SNPs [rs17374292 (*PER3*); rs6722909, rs12712085 (*NPAS2*); rs7950226, rs2278749 (*ARNTL*); rs7137588, rs17413842 (*ARNTL2*)] failed the Hardy-Weinberg equilibrium test and were excluded from further analyses.

The second study sample was genotyped with Illumina 610 K platform (Illumina Inc. San Diego, CA, USA). The call rate was >95% both for individuals and markers. The markers with MAF <1% or Hardy-Weinberg p <1×10^−6^ had been excluded.

### Statistical Analyses

We compared the allele frequencies between cases and controls using chi-square tests as implemented in the PLINK software package, web-based version 1.06 (http://pngu.mgh.harvard.edu/purcell/plink/) [82]. To exclude possible false-positive results, PLINK's *max (T)* permutation test with 10,000 permutations was used to generate empirical p-values and for multiple testing correction. Power calculations show that our sample was powered (Table S6) to detect associations for variants that increase risk depression ≥1.5 fold in females and ≥1.8 fold in males at 〈 = 0.05 and ® = 0.80 and ≥2.0 fold in females and ≥2.7 fold in males at 〈 = 0.05/(2×113×3)  = 0.000073 threshold level for statistical significance when taken into account the number of tests and ® = 0.80.

In the single-locus analysis, we compared the following groups: (1) all depressed patients against all controls (D+ vs. D–), (2) depressed patients with early morning awakening against controls without early morning awakening (D+EMA+ vs. D–EMA–), and (3) depressed patients with fatigue against controls without fatigue (D+FAT+ vs. D–FAT–). To check gender-dependent and symptom-specific differences in the genetic background of depression, females and males were analyzed separately.

Subsequently, we performed a descriptive analysis and compared allelic frequencies of gene variants that gave any evidence for an association (P<0.05, not corrected for multiple testing). The following non-overlapping groups were analyzed: (1) D+EMA–FAT– (n = 41 females and 16 males), (2) D+EMA+FAT+ (n = 94 females and 58 males), (3) D+EMA+FAT– (n = 15 females and 3 males; owing to the small number of males in this group, we did not examine their allelic frequencies), (4) D+EMA–FAT+ (n = 91 females and 33 males), and (5) controls, D–EMA–FAT– (n = 578 females and 481 males).

Factors 1 and 2 from GSS were analyzed using linear regression models including age and affection status as covariates. This model was constructed separately for all females (n = 967) and all males (n = 687). We also implemented similar type of analyses for second sample set in which the status for metabolic disorder as well as age were the covariates.

We also used Haploview (V.4.1) to determine the pair-wise LD structure for all genotyped variations within each studied circadian gene [81]. We then performed two-SNP, three-SNP and four-SNP haplotype association analyses by utilizing SNPs in genes giving an association of P<0.05 in the single-locus analyses of the first sample set.

Furthermore, the logistic regression model, as implemented in the PLINK software package (V.1.06) [82], was used to investigate interaction of gender between variants of most significantly associated gene and study phenotypes, also used to SNP-SNP interaction analyses for SNPs that gave significant results when comparing D+FAT+ females vs. D–FAT– females, and D+EMA+ males vs. D–EMA– males.

Finally, to search for transcription factor binding sites within a particular *TIMELESS* gene that was differentially associated with both genders for depression and sleep-related problems, we implemented the tool ConSite, a platform-independent web resource [83]. The corresponding regulatory regions of human (ENSG00000111602) and target mouse (ENSMUSG00000039994) were retrieved using a genome browser such as EnsEMBL (www.ensembl.org), and the retrieved orthologous pairs of genomic sequences were re-aligned using the ORCA aligner [83]. We then examined the transcription factor binding sites shared by this gene. Only vertebrate transcription factors, with a specificity of minimum 10 bits and a TF score threshold of 90%, in parts of the sequences presenting a minimum conservation of 90% between the species.

## Supporting Information

Figure S1 Linkage disequilibrium (LD) structures of PER3 SNPs. LD structures of PER3 SNPs based on (A) the Health 2000 study sample, (B) based on the Health 2000 sub sample that was genotyped for genome wide association study (GWA), and (C) based on the HapMap data with all possible SNPs found in (A),(B) and SNPs rs10462020, rs2640909, rs10462021 studied by Ebisawa et al., (EMBO reports 2001:2:342).
(7.80 MB TIF)

Figure S2 Geographical characteristics of the study samples. The Health 2000 cohort was collected from 5 university hospital areas, 1) Helsinki and Uusimaa, 2) Varsinais-Suomi, 3) Pirkanmaa, 4) Pohjois-Savo, and 5) Pohjois-Pohjanmaa; red, green color marked for cases and controls, respectively.
(1.79 MB TIF)

Table S1 Complete results for analysis between depressive disorder and SNPs of the circadian clock genes in females from Finnish population. aSNPs yielding permutated pointwise p-values, bold are SNPs having the p-values < 0.05 in the single-locus analyses. bAlleles: Major/Minor. D+, patients with depression. D-, controls (no depression). D+EMA+, depressed patients with early morning awakening. D-EMA-, controls without early morning awakening. D+FAT+, depressed patients with fatigue. D-FAT-, controls without fatigue. *SNP indicated permutation-based corrected empirical P-value (P<0.05) over the model D+FAT+ vs.D-FAT- .
(0.05 MB XLS)

Table S2 Complete results for analysis between depressive disorder and SNPs of the circadian clock genes in males from Finnish population. aSNPs yielding permutated pointwise p-values, bold are SNPs having the p-values < 0.05 in the single-locus analyses. bAlleles: Major/Minor. D+, patients with depression. D-, controls (no depression). D+EMA+, depressed patients with early morning awakening. D-EMA-, controls without early morning awakening. D+FAT+, depressed patients with fatigue. D-FAT-, controls without fatigue. *SNP indicated permutation-based corrected empirical P-value (P<0.05) over the model D+EMA+ vs. D-EMA-.
(0.04 MB XLS)

Table S3 Results from association analysis of Global Seasonality Score (GSS) with the sample of depressed females and males. SNPs yielding suggestive P-values (P<0.05). Alleles: Major/Minor. β = Regression coefficient. P-values for the GSS factor 1 and factor 2 were generated using the linear regression model including age and affection status as covariates.
(0.02 MB XLS)

Table S4 Linkage Disequilibrium (LD) patterns for all genotyped variations within each of the studied circadian genes in the Health 2000 dataset.
(1.00 MB DOC)

Table S5 List of genotyped circadian SNPs in the current study. The variant information is from the NCBI dbSNP BUILD 125 and 129 (http://www.ncbi.nlm.nih.gov/).
(0.03 MB XLS)

Table S6 Power calculations.
(0.03 MB XLS)
