# Supplementary figures and images for: Systematic Analysis of Circadian Genes in a Population-Based Sample Reveals Association of TIMELESS with Depression and Sleep Disturbance
Source: PLoS One. 2010 Feb 18;5(2):e9259. doi: 10.1371/journal.pone.0009259 (PMC2823770; doi:10.1371/journal.pone.0009259)

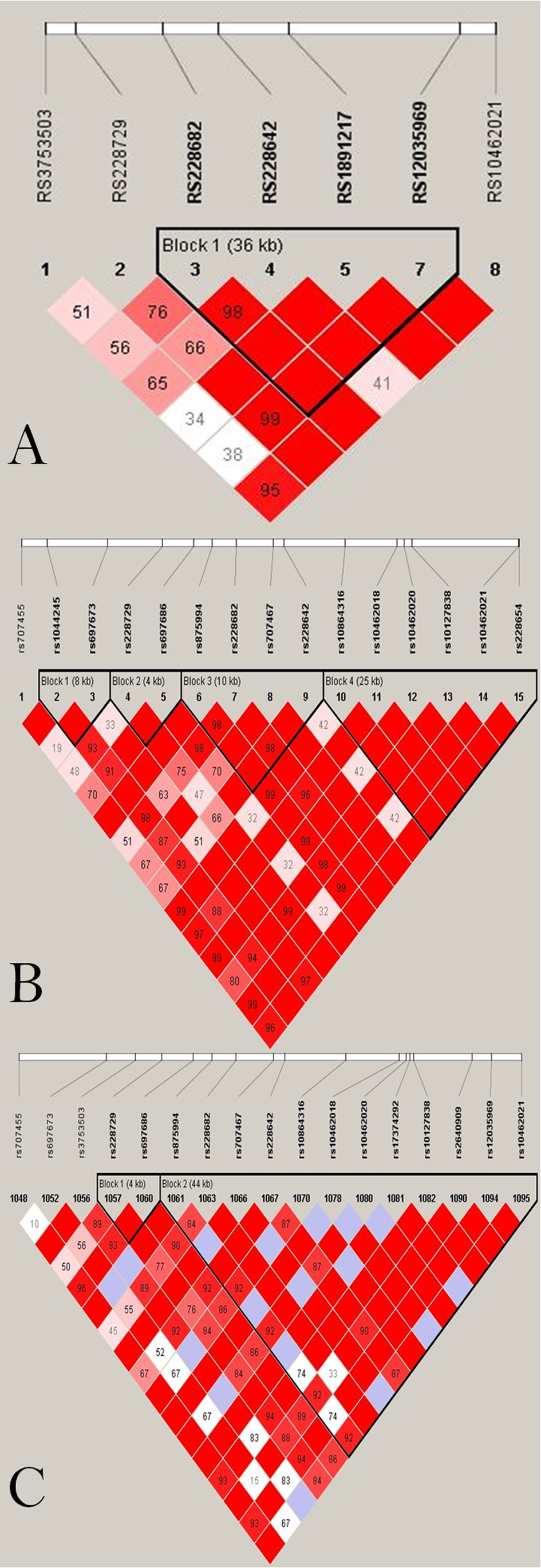

Supplement: Figure S1 — Linkage disequilibrium (LD) structures of PER3 SNPs. LD structures of PER3 SNPs based on (A) the Health 2000 study sample, (B) based on the Health 2000 sub sample that was genotyped for genome wide association study (GWA), and (C) based on the HapMap data with all possible SNPs found in (A),(B) and SNPs rs10462020, rs2640909, rs10462021 studied by Ebisawa et al., (EMBO reports 2001:2:342). (7.80 MB TIF) [file pone.0009259.s001.tif]

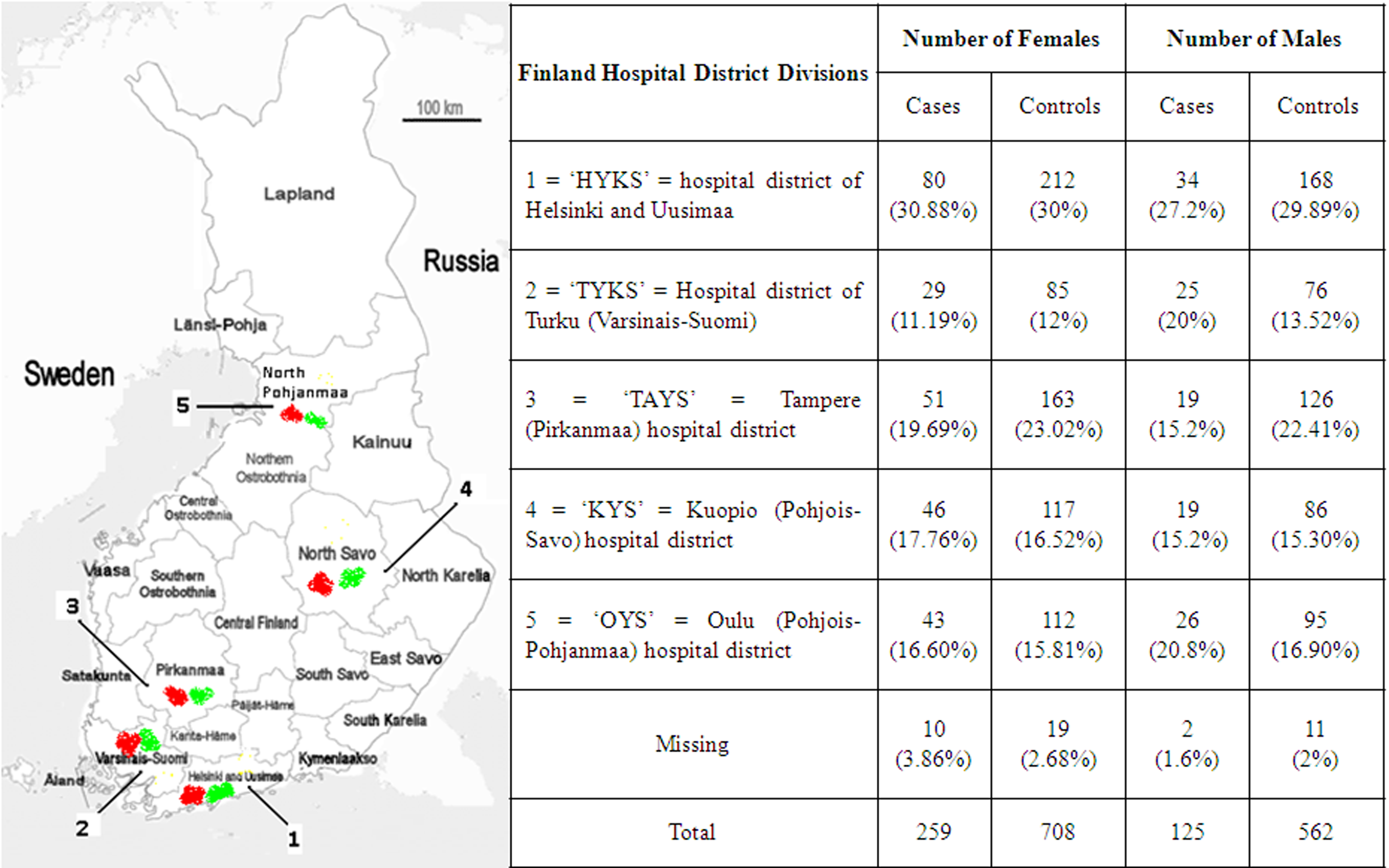

Supplement: Figure S2 — Geographical characteristics of the study samples. The Health 2000 cohort was collected from 5 university hospital areas, 1) Helsinki and Uusimaa, 2) Varsinais-Suomi, 3) Pirkanmaa, 4) Pohjois-Savo, and 5) Pohjois-Pohjanmaa; red, green color marked for cases and controls, respectively. (1.79 MB TIF) [file pone.0009259.s002.tif]
