# Supplementary material for: Systematic Analysis of Circadian Genes in a Population-Based Sample Reveals Association of TIMELESS with Depression and Sleep Disturbance
Source: PLoS One. 2010 Feb 18;5(2):e9259. doi: 10.1371/journal.pone.0009259 (PMC2823770; doi:10.1371/journal.pone.0009259)

**Table S4. Linkage Disequilibrium (LD) patterns for all genotyped variations within each of the studied circadian genes in the Health 2000 dataset.**

| Table 1: SNPs analyzed in *PER3* gene | | | | | | | | |
| --- | --- | --- | --- | --- | --- | --- | --- | --- |
| L1 | L2 | D' | LOD | r^2 | CIlow | CIhi | Dist | T-int |
| RS3753503 | RS228729 | 0.517 | 4.97 | 0.013 | 0.34 | 0.66 | 3724 | 33.46 |
| RS3753503 | RS228682 | 0.568 | 6.91 | 0.019 | 0.4 | 0.7 | 14375 | - |
| RS3753503 | RS228642 | 0.653 | 19.55 | 0.052 | 0.54 | 0.74 | 21322 | - |
| RS3753503 | RS1891217 | 0.34 | 0.18 | 0.0010 | 0.04 | 0.85 | 29878 | - |
| RS3753503 | RS12035969 | 0.38 | 1.85 | 0.0040 | 0.16 | 0.56 | 50875 | - |
| RS3753503 | RS10462021 | 0.957 | 13.31 | 0.028 | 0.81 | 0.99 | 55162 | - |
| RS228729 | RS228682 | 0.76 | 228.54 | 0.484 | 0.72 | 0.79 | 10651 | 813.42 |
| RS228729 | RS228642 | 0.66 | 74.28 | 0.179 | 0.6 | 0.71 | 17598 | - |
| RS228729 | RS1891217 | 1.0 | 47.09 | 0.104 | 0.96 | 1.0 | 26154 | - |
| RS228729 | RS12035969 | 0.998 | 329.18 | 0.605 | 0.98 | 1.0 | 47151 | - |
| RS228729 | RS10462021 | 1.0 | 92.53 | 0.175 | 0.98 | 1.0 | 51438 | - |
| RS228682 | RS228642 | 0.989 | 267.91 | 0.48 | 0.97 | 1.0 | 6947 | 1277.98 |
| RS228682 | RS1891217 | 1.0 | 41.52 | 0.087 | 0.95 | 1.0 | 15503 | - |
| RS228682 | RS12035969 | 1.0 | 279.7 | 0.51 | 0.99 | 1.0 | 36500 | - |
| RS228682 | RS10462021 | 1.0 | 110.88 | 0.207 | 0.98 | 1.0 | 40787 | - |
| RS228642 | RS1891217 | 1.0 | 18.83 | 0.042 | 0.9 | 1.0 | 8556 | 1090.04 |
| RS228642 | RS12035969 | 1.0 | 138.45 | 0.251 | 0.98 | 1.0 | 29553 | - |
| RS228642 | RS10462021 | 0.415 | 16.52 | 0.043 | 0.33 | 0.49 | 33840 | - |
| RS1891217 | RS12035969 | 1.0 | 8.88 | 0.018 | 0.79 | 1.0 | 20997 | 1003.4 |
| RS1891217 | RS10462021 | 1.0 | 12.1 | 0.018 | 0.86 | 1.0 | 25284 | - |
| RS12035969 | RS10462021 | 1.0 | 51.94 | 0.106 | 0.96 | 1.0 | 4287 | 283.97 |

Figure 1. Haploview LD display of *PER3* SNPs


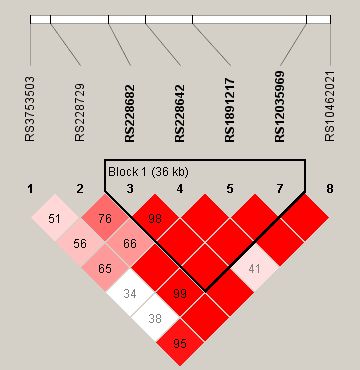


| Table 2: SNPs analyzed in *PER2* gene | | | | | | | | |
| --- | --- | --- | --- | --- | --- | --- | --- | --- |
| L1 | L2 | D' | LOD | r^2 | CIlow | CIhi | Dist | T-int |
| RS881933 | RS934945 | 0.809 | 84.71 | 0.227 | 0.75 | 0.86 | 4012 | 272.8 |
| RS881933 | RS6431590 | 0.322 | 18.49 | 0.048 | 0.26 | 0.38 | 14087 | - |
| RS881933 | RS3739064 | 0.297 | 26.31 | 0.068 | 0.24 | 0.35 | 25345 | - |
| RS881933 | RS11894535 | 0.476 | 86.49 | 0.22 | 0.43 | 0.52 | 26032 | - |
| RS881933 | RS10462023 | 0.809 | 56.8 | 0.13 | 0.74 | 0.86 | 33540 | - |
| RS881933 | RS2304672 | 1.0 | 44.63 | 0.078 | 0.96 | 1.0 | 35548 | - |
| RS881933 | RS4663302 | 0.127 | 4.9 | 0.014 | 0.07 | 0.18 | 52720 | - |
| RS934945 | RS6431590 | 1.0 | 59.38 | 0.107 | 0.97 | 1.0 | 10075 | 376.36 |
| RS934945 | RS3739064 | 0.969 | 34.17 | 0.061 | 0.9 | 1.0 | 21333 | - |
| RS934945 | RS11894535 | 0.955 | 23.25 | 0.045 | 0.86 | 0.99 | 22020 | - |
| RS934945 | RS10462023 | 0.681 | 10.72 | 0.032 | 0.53 | 0.79 | 29528 | - |
| RS934945 | RS2304672 | 1.0 | 16.12 | 0.027 | 0.88 | 1.0 | 31536 | - |
| RS934945 | RS4663302 | 0.876 | 119.08 | 0.299 | 0.83 | 0.92 | 48708 | - |
| RS6431590 | RS3739064 | 0.981 | 326.07 | 0.579 | 0.96 | 1.0 | 11258 | 1398.17 |
| RS6431590 | RS11894535 | 0.977 | 227.92 | 0.432 | 0.95 | 0.99 | 11945 | - |
| RS6431590 | RS10462023 | 0.917 | 173.98 | 0.357 | 0.88 | 0.95 | 19453 | - |
| RS6431590 | RS2304672 | 1.0 | 130.05 | 0.251 | 0.98 | 1.0 | 21461 | - |
| RS6431590 | RS4663302 | 0.931 | 117.68 | 0.242 | 0.89 | 0.96 | 38633 | - |
| RS3739064 | RS11894535 | 0.375 | 40.91 | 0.105 | 0.32 | 0.43 | 687 | 1491.52 |
| RS3739064 | RS10462023 | 1.0 | 142.34 | 0.255 | 0.98 | 1.0 | 8195 | - |
| RS3739064 | RS2304672 | 1.0 | 213.69 | 0.42 | 0.99 | 1.0 | 10203 | - |
| RS3739064 | RS4663302 | 0.975 | 82.96 | 0.159 | 0.93 | 1.0 | 27375 | - |
| RS11894535 | RS10462023 | 0.951 | 86.42 | 0.175 | 0.91 | 0.98 | 7508 | 1304.51 |
| RS11894535 | RS2304672 | 1.0 | 40.73 | 0.077 | 0.95 | 1.0 | 9516 | - |
| RS11894535 | RS4663302 | 1.0 | 64.41 | 0.126 | 0.97 | 1.0 | 26688 | - |
| RS10462023 | RS2304672 | 1.0 | 52.47 | 0.107 | 0.96 | 1.0 | 2008 | 866.97 |
| RS10462023 | RS4663302 | 0.659 | 29.78 | 0.077 | 0.57 | 0.73 | 19180 | - |
| RS2304672 | RS4663302 | 0.968 | 32.93 | 0.066 | 0.89 | 1.0 | 17172 | 327.76 |

Figure 2. Haploview LD display of *PER2* SNPs


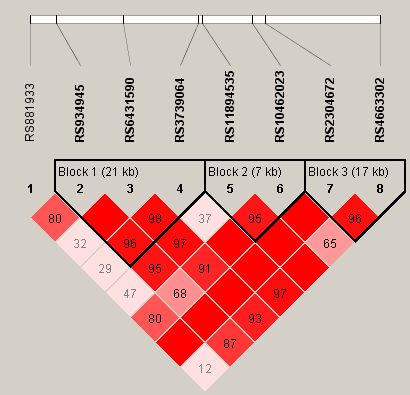


| Table 3: SNPs analyzed in *NPAS2* gene | | | | | | | | |
| --- | --- | --- | --- | --- | --- | --- | --- | --- |
| L1 | L2 | D' | LOD | r^2 | CIlow | CIhi | Dist | T-int |
| RS1811399 | RS12712083 | 0.303 | 6.72 | 0.019 | 0.2 | 0.39 | 29885 | 65.7 |
| RS1811399 | RS2117714 | 0.068 | 0.97 | 0.0030 | 0.01 | 0.13 | 42763 | - |
| RS1811399 | RS6725296 | 0.309 | 17.22 | 0.052 | 0.24 | 0.37 | 58690 | - |
| RS1811399 | RS3820785 | 0.354 | 39.04 | 0.108 | 0.3 | 0.4 | 68800 | - |
| RS1811399 | RS17025078 | 0.302 | 1.75 | 0.0050 | 0.12 | 0.46 | 100562 | - |
| RS1811399 | RS2305160 | 0.094 | 0.36 | 0.0010 | 0.0 | 0.22 | 112290 | - |
| RS1811399 | S471L | 0.331 | 2.5 | 0.0060 | 0.16 | 0.48 | 115177 | - |
| RS1811399 | RS1374324 | 0.297 | 12.48 | 0.034 | 0.22 | 0.37 | 135308 | - |
| RS12712083 | RS2117714 | 0.832 | 188.81 | 0.404 | 0.79 | 0.87 | 12878 | 359.77 |
| RS12712083 | RS6725296 | 0.876 | 68.52 | 0.152 | 0.82 | 0.92 | 28805 | - |
| RS12712083 | RS3820785 | 0.377 | 22.62 | 0.06 | 0.31 | 0.44 | 38915 | - |
| RS12712083 | RS17025078 | 0.563 | 16.93 | 0.045 | 0.46 | 0.65 | 70677 | - |
| RS12712083 | RS2305160 | 0.178 | 3.55 | 0.0090 | 0.1 | 0.26 | 82405 | - |
| RS12712083 | S471L | 0.241 | 3.27 | 0.0090 | 0.13 | 0.35 | 85292 | - |
| RS12712083 | RS1374324 | 0.085 | 2.42 | 0.0070 | 0.03 | 0.14 | 105423 | - |
| RS2117714 | RS6725296 | 0.957 | 136.87 | 0.31 | 0.92 | 0.98 | 15927 | 342.87 |
| RS2117714 | RS3820785 | 0.149 | 1.12 | 0.0030 | 0.04 | 0.26 | 26037 | - |
| RS2117714 | RS17025078 | 0.406 | 5.02 | 0.014 | 0.26 | 0.53 | 57799 | - |
| RS2117714 | RS2305160 | 0.575 | 22.74 | 0.056 | 0.48 | 0.66 | 69527 | - |
| RS2117714 | S471L | 0.212 | 1.36 | 0.0040 | 0.07 | 0.35 | 72414 | - |
| RS2117714 | RS1374324 | 0.152 | 5.01 | 0.014 | 0.09 | 0.21 | 92545 | - |
| RS6725296 | RS3820785 | 0.3 | 14.7 | 0.042 | 0.23 | 0.37 | 10110 | 181.11 |
| RS6725296 | RS17025078 | 0.84 | 8.45 | 0.02 | 0.64 | 0.94 | 41872 | - |
| RS6725296 | RS2305160 | 0.643 | 10.46 | 0.024 | 0.5 | 0.75 | 53600 | - |
| RS6725296 | S471L | 0.779 | 6.26 | 0.018 | 0.54 | 0.91 | 56487 | - |
| RS6725296 | RS1374324 | 0.118 | 1.07 | 0.0030 | 0.03 | 0.21 | 76618 | - |
| RS3820785 | RS17025078 | 0.0030 | 0.0 | 0.0 | -0.01 | 0.07 | 31762 | 139.01 |
| RS3820785 | RS2305160 | 0.077 | 1.77 | 0.0050 | 0.02 | 0.13 | 43490 | - |
| RS3820785 | S471L | 0.564 | 8.53 | 0.02 | 0.42 | 0.68 | 46377 | - |
| RS3820785 | RS1374324 | 0.394 | 25.08 | 0.069 | 0.33 | 0.46 | 66508 | - |
| RS17025078 | RS2305160 | 0.97 | 34.8 | 0.069 | 0.9 | 1.0 | 11728 | 183.56 |
| RS17025078 | S471L | 1.0 | 21.84 | 0.038 | 0.91 | 1.0 | 14615 | - |
| RS17025078 | RS1374324 | 0.575 | 35.4 | 0.089 | 0.5 | 0.64 | 34746 | - |
| RS2305160 | S471L | 1.0 | 42.17 | 0.078 | 0.95 | 1.0 | 2887 | 147.04 |
| RS2305160 | RS1374324 | 0.058 | 0.32 | 0.0010 | 0.0 | 0.15 | 23018 | - |
| S471L | RS1374324 | 0.835 | 45.17 | 0.099 | 0.76 | 0.89 | 20131 | 107.04 |

Figure 3. Haploview LD display of *NPAS2* SNPs


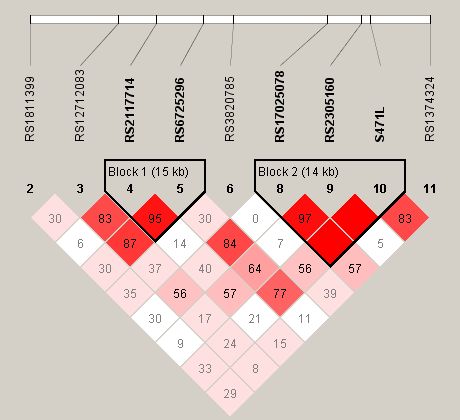


| Table 4: SNPs analyzed in *CLOCK* gene | | | | | | | | |
| --- | --- | --- | --- | --- | --- | --- | --- | --- |
| L1 | L2 | D' | LOD | r^2 | CIlow | CIhi | Dist | T-int |
| RS10462028 | RS1801260 | 1.0 | 428.75 | 0.821 | 0.99 | 1.0 | 3069 | 736.29 |
| RS10462028 | RS11932595 | 0.747 | 189.71 | 0.404 | 0.71 | 0.78 | 25297 | - |
| RS10462028 | RS6850524 | 0.586 | 37.67 | 0.096 | 0.51 | 0.65 | 83697 | - |
| RS10462028 | RS4864548 | 0.681 | 80.16 | 0.178 | 0.63 | 0.73 | 115503 | - |
| RS1801260 | RS11932595 | 0.959 | 251.74 | 0.547 | 0.93 | 0.98 | 22228 | 732.08 |
| RS1801260 | RS6850524 | 0.521 | 19.52 | 0.062 | 0.43 | 0.6 | 80628 | - |
| RS1801260 | RS4864548 | 1.0 | 153.28 | 0.313 | 0.98 | 1.0 | 112434 | - |
| RS11932595 | RS6850524 | 0.664 | 70.73 | 0.17 | 0.61 | 0.72 | 58400 | 375.36 |
| RS11932595 | RS4864548 | 0.26 | 14.0 | 0.036 | 0.2 | 0.32 | 90206 | - |
| RS6850524 | RS4864548 | 1.0 | 187.12 | 0.32 | 0.99 | 1.0 | 31806 | 434.56 |

Figure 4. Haploview LD display of *CLOCK* SNPs


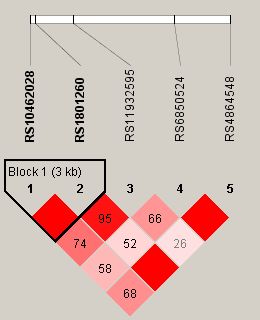


| Table 5: SNPs analyzed in *NFIL3* gene | | | | | | | | |
| --- | --- | --- | --- | --- | --- | --- | --- | --- |
| L1 | L2 | D' | LOD | r^2 | CIlow | CIhi | Dist | T-int |
| RS1619450 | RS10991925 | 1.0 | 16.0 | 0.03 | 0.88 | 1.0 | 8485 | 139.38 |
| RS1619450 | RS2440589 | 1.0 | 103.37 | 0.209 | 0.98 | 1.0 | 9832 | - |
| RS1619450 | RS968821 | 0.97 | 19.16 | 0.039 | 0.86 | 1.0 | 18479 | - |
| RS1619450 | RS813498 | 1.0 | 0.85 | 0.0020 | 0.14 | 0.99 | 19231 | - |
| RS10991925 | RS2440589 | 1.0 | 223.21 | 0.424 | 0.99 | 1.0 | 1347 | 693.94 |
| RS10991925 | RS968821 | 0.991 | 345.46 | 0.699 | 0.97 | 1.0 | 9994 | - |
| RS10991925 | RS813498 | 1.0 | 1.89 | 0.0040 | 0.35 | 1.0 | 10746 | - |
| RS2440589 | RS968821 | 1.0 | 331.96 | 0.599 | 0.99 | 1.0 | 8647 | 704.13 |
| RS2440589 | RS813498 | 1.0 | 4.81 | 0.0090 | 0.67 | 1.0 | 9399 | - |
| RS968821 | RS813498 | 1.0 | 2.88 | 0.0050 | 0.5 | 1.0 | 752 | 10.43 |

Figure 5. Haploview LD display of *NFIL3* SNPs


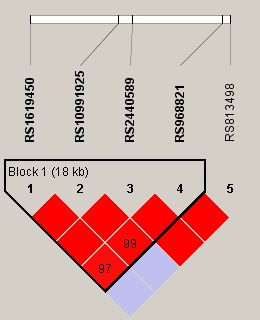


| Table 6: SNPs analyzed in *BHLHE40* gene | | | | | | | | |
| --- | --- | --- | --- | --- | --- | --- | --- | --- |
| L1 | L2 | D' | LOD | r^2 | CIlow | CIhi | Dist | T-int |
| RS908078 | RS11130215 | 0.929 | 232.34 | 0.51 | 0.9 | 0.96 | 1237 | 252.78 |
| RS908078 | RS2137947 | 0.891 | 20.44 | 0.046 | 0.77 | 0.95 | 6190 | - |
| RS11130215 | RS2137947 | 0.977 | 46.25 | 0.094 | 0.92 | 1.0 | 4953 | 66.69 |

Figure 6. Haploview LD display of *BHLHE40* SNPs


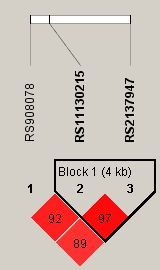


| Table 7: SNPs analyzed in *CRY2* gene | | | | | | | | |
| --- | --- | --- | --- | --- | --- | --- | --- | --- |
| L1 | L2 | D' | LOD | r^2 | CIlow | CIhi | Dist | T-int |
| RS10838524 | RS7123390 | 0.883 | 126.46 | 0.285 | 0.84 | 0.92 | 21241 | 367.46 |
| RS10838524 | RS10838527 | 1.0 | 66.36 | 0.125 | 0.97 | 1.0 | 33017 | - |
| RS10838524 | RS3824872 | 1.0 | 174.64 | 0.313 | 0.98 | 1.0 | 35428 | - |
| RS7123390 | RS10838527 | 1.0 | 26.41 | 0.051 | 0.93 | 1.0 | 11776 | 334.87 |
| RS7123390 | RS3824872 | 1.0 | 67.46 | 0.128 | 0.97 | 1.0 | 14187 | - |
| RS10838527 | RS3824872 | 1.0 | 185.59 | 0.397 | 0.98 | 1.0 | 2411 | 427.69 |

Figure 7. Haploview LD display of *CRY2* SNPs


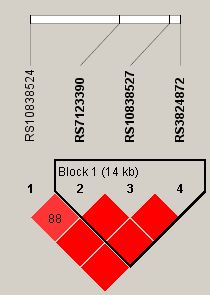


| Table 8: SNPs analyzed in *ARNTL* gene | | | | | | | | |
| --- | --- | --- | --- | --- | --- | --- | --- | --- |
| L1 | L2 | D' | LOD | r^2 | CIlow | CIhi | Dist | T-int |
| RS2279287 | RS10766074 | 0.801 | 102.85 | 0.247 | 0.75 | 0.85 | 20081 | 389.55 |
| RS2279287 | RS1982350 | 0.609 | 84.41 | 0.199 | 0.56 | 0.66 | 51646 | - |
| RS2279287 | RS6486121 | 0.639 | 72.82 | 0.175 | 0.58 | 0.69 | 57285 | - |
| RS2279287 | RS1562438 | 0.578 | 121.64 | 0.293 | 0.54 | 0.62 | 65715 | - |
| RS2279287 | RS2290036 | 0.607 | 7.83 | 0.021 | 0.44 | 0.73 | 81303 | - |
| RS2279287 | RS1868049 | 0.127 | 2.28 | 0.0070 | 0.05 | 0.2 | 85197 | - |
| RS2279287 | RS11022778 | 0.038 | 0.44 | 0.0010 | -0.01 | 0.09 | 92375 | - |
| RS2279287 | RS3816358 | 0.07 | 0.62 | 0.0020 | 0.0 | 0.15 | 92987 | - |
| RS2279287 | RS4757151 | 0.151 | 3.07 | 0.0090 | 0.08 | 0.22 | 93728 | - |
| RS2279287 | RS3897902 | 0.174 | 3.49 | 0.01 | 0.09 | 0.25 | 103318 | - |
| RS2279287 | RS969485 | 0.073 | 1.24 | 0.0030 | 0.01 | 0.13 | 104558 | - |
| RS10766074 | RS1982350 | 0.896 | 77.54 | 0.166 | 0.84 | 0.94 | 31565 | 490.68 |
| RS10766074 | RS6486121 | 0.762 | 39.0 | 0.096 | 0.68 | 0.83 | 37204 | - |
| RS10766074 | RS1562438 | 0.776 | 79.78 | 0.202 | 0.72 | 0.83 | 45634 | - |
| RS10766074 | RS2290036 | 0.743 | 5.22 | 0.012 | 0.5 | 0.88 | 61222 | - |
| RS10766074 | RS1868049 | 0.116 | 0.16 | 0.0 | 0.0 | 0.35 | 65116 | - |
| RS10766074 | RS11022778 | 0.143 | 2.98 | 0.0090 | 0.07 | 0.22 | 72294 | - |
| RS10766074 | RS3816358 | 0.322 | 1.12 | 0.0030 | 0.09 | 0.53 | 72906 | - |
| RS10766074 | RS4757151 | 0.185 | 1.76 | 0.0050 | 0.07 | 0.3 | 73647 | - |
| RS10766074 | RS3897902 | 0.097 | 0.09 | 0.0 | 0.0 | 0.35 | 83237 | - |
| RS10766074 | RS969485 | 0.229 | 1.0 | 0.0030 | 0.05 | 0.4 | 84477 | - |
| RS1982350 | RS6486121 | 0.723 | 194.02 | 0.417 | 0.69 | 0.76 | 5639 | 635.82 |
| RS1982350 | RS1562438 | 0.642 | 106.45 | 0.25 | 0.59 | 0.69 | 14069 | - |
| RS1982350 | RS2290036 | 0.166 | 1.46 | 0.0040 | 0.05 | 0.28 | 29657 | - |
| RS1982350 | RS1868049 | 0.028 | 0.04 | 0.0 | -0.01 | 0.15 | 33551 | - |
| RS1982350 | RS11022778 | 0.1 | 1.7 | 0.0050 | 0.03 | 0.17 | 40729 | - |
| RS1982350 | RS3816358 | 0.481 | 16.89 | 0.044 | 0.39 | 0.57 | 41341 | - |
| RS1982350 | RS4757151 | 0.199 | 10.68 | 0.03 | 0.14 | 0.25 | 42082 | - |
| RS1982350 | RS3897902 | 0.04 | 0.1 | 0.0 | -0.01 | 0.15 | 51672 | - |
| RS1982350 | RS969485 | 0.141 | 1.71 | 0.0050 | 0.05 | 0.23 | 52912 | - |
| RS6486121 | RS1562438 | 0.929 | 223.65 | 0.443 | 0.9 | 0.95 | 8430 | 594.83 |
| RS6486121 | RS2290036 | 0.197 | 1.56 | 0.0050 | 0.07 | 0.32 | 24018 | - |
| RS6486121 | RS1868049 | 0.07 | 0.32 | 0.0010 | 0.0 | 0.18 | 27912 | - |
| RS6486121 | RS11022778 | 0.165 | 3.83 | 0.01 | 0.09 | 0.24 | 35090 | - |
| RS6486121 | RS3816358 | 0.54 | 16.86 | 0.044 | 0.44 | 0.63 | 35702 | - |
| RS6486121 | RS4757151 | 0.16 | 8.38 | 0.024 | 0.11 | 0.21 | 36443 | - |
| RS6486121 | RS3897902 | 0.19 | 2.07 | 0.0060 | 0.08 | 0.3 | 46033 | - |
| RS6486121 | RS969485 | 0.26 | 7.59 | 0.02 | 0.18 | 0.34 | 47273 | - |
| RS1562438 | RS2290036 | 0.605 | 8.03 | 0.024 | 0.44 | 0.73 | 15588 | 142.82 |
| RS1562438 | RS1868049 | 0.46 | 7.46 | 0.019 | 0.33 | 0.58 | 19482 | - |
| RS1562438 | RS11022778 | 0.261 | 18.94 | 0.053 | 0.21 | 0.31 | 26660 | - |
| RS1562438 | RS3816358 | 0.107 | 1.26 | 0.0040 | 0.03 | 0.19 | 27272 | - |
| RS1562438 | RS4757151 | 0.352 | 19.93 | 0.056 | 0.28 | 0.42 | 28013 | - |
| RS1562438 | RS3897902 | 0.167 | 0.78 | 0.0020 | 0.03 | 0.31 | 37603 | - |
| RS1562438 | RS969485 | 0.184 | 1.9 | 0.0050 | 0.07 | 0.29 | 38843 | - |
| RS2290036 | RS1868049 | 1.0 | 9.44 | 0.023 | 0.78 | 1.0 | 3894 | 292.06 |
| RS2290036 | RS11022778 | 0.78 | 10.8 | 0.031 | 0.61 | 0.88 | 11072 | - |
| RS2290036 | RS3816358 | 0.7 | 129.08 | 0.403 | 0.65 | 0.75 | 11684 | - |
| RS2290036 | RS4757151 | 0.7 | 20.82 | 0.057 | 0.59 | 0.78 | 12425 | - |
| RS2290036 | RS3897902 | 1.0 | 6.57 | 0.02 | 0.69 | 1.0 | 22015 | - |
| RS2290036 | RS969485 | 1.0 | 16.14 | 0.037 | 0.88 | 1.0 | 23255 | - |
| RS1868049 | RS11022778 | 1.0 | 41.25 | 0.072 | 0.95 | 1.0 | 7178 | 725.69 |
| RS1868049 | RS3816358 | 1.0 | 14.87 | 0.029 | 0.87 | 1.0 | 7790 | - |
| RS1868049 | RS4757151 | 0.871 | 69.02 | 0.154 | 0.81 | 0.92 | 8531 | - |
| RS1868049 | RS3897902 | 0.803 | 196.41 | 0.548 | 0.76 | 0.84 | 18121 | - |
| RS1868049 | RS969485 | 0.685 | 108.11 | 0.29 | 0.63 | 0.73 | 19361 | - |
| RS11022778 | RS3816358 | 0.827 | 16.77 | 0.043 | 0.69 | 0.91 | 612 | 774.74 |
| RS11022778 | RS4757151 | 0.85 | 118.04 | 0.256 | 0.8 | 0.89 | 1353 | - |
| RS11022778 | RS3897902 | 0.642 | 11.3 | 0.025 | 0.5 | 0.75 | 10943 | - |
| RS11022778 | RS969485 | 0.182 | 8.84 | 0.024 | 0.13 | 0.24 | 12183 | - |
| RS3816358 | RS4757151 | 0.793 | 35.64 | 0.09 | 0.71 | 0.86 | 741 | 646.89 |
| RS3816358 | RS3897902 | 1.0 | 10.34 | 0.024 | 0.8 | 1.0 | 10331 | - |
| RS3816358 | RS969485 | 1.0 | 23.05 | 0.046 | 0.91 | 1.0 | 11571 | - |
| RS4757151 | RS3897902 | 0.959 | 73.67 | 0.158 | 0.91 | 0.99 | 9590 | 459.82 |
| RS4757151 | RS969485 | 0.215 | 5.39 | 0.015 | 0.13 | 0.29 | 10830 | - |
| RS3897902 | RS969485 | 0.973 | 215.82 | 0.509 | 0.94 | 0.99 | 1240 | 361.21 |

Figure 8. Haploview LD display of *ARNTL* SNPs


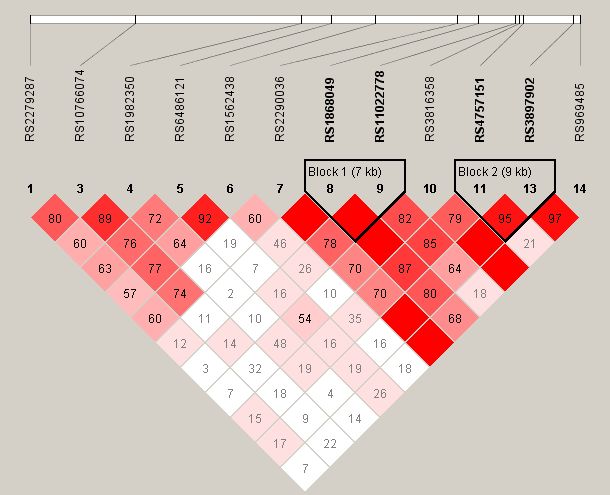


| Table 9: SNPs analyzed in *ARNTL2* gene | | | | | | | | |
| --- | --- | --- | --- | --- | --- | --- | --- | --- |
| L1 | L2 | D' | LOD | r^2 | CIlow | CIhi | Dist | T-int |
| RS10842905 | RS4964052 | 0.76 | 94.77 | 0.219 | 0.71 | 0.81 | 6427 | 177.29 |
| RS10842905 | RS922270 | 0.72 | 47.39 | 0.121 | 0.65 | 0.78 | 22048 | - |
| RS10842905 | RS4964060 | 0.407 | 25.81 | 0.068 | 0.34 | 0.47 | 50464 | - |
| RS10842905 | RS7304939 | 0.293 | 1.79 | 0.0050 | 0.12 | 0.45 | 61442 | - |
| RS10842905 | RS12299407 | 0.441 | 7.53 | 0.02 | 0.31 | 0.55 | 66280 | - |
| RS10842905 | RS1037921 | 0.595 | 6.23 | 0.015 | 0.41 | 0.73 | 70663 | - |
| RS10842905 | RS4931075 | 0.681 | 40.29 | 0.098 | 0.6 | 0.75 | 82243 | - |
| RS10842905 | RS2289709 | 0.011 | 0.01 | 0.0 | -0.01 | 0.11 | 90730 | - |
| RS4964052 | RS922270 | 1.0 | 47.92 | 0.089 | 0.96 | 1.0 | 15621 | 158.99 |
| RS4964052 | RS4964060 | 0.171 | 6.39 | 0.017 | 0.11 | 0.23 | 44037 | - |
| RS4964052 | RS7304939 | 0.507 | 7.66 | 0.019 | 0.36 | 0.63 | 55015 | - |
| RS4964052 | RS12299407 | 0.162 | 2.7 | 0.0070 | 0.08 | 0.25 | 59853 | - |
| RS4964052 | RS1037921 | 0.487 | 5.57 | 0.014 | 0.32 | 0.62 | 64236 | - |
| RS4964052 | RS4931075 | 0.066 | 0.97 | 0.0020 | 0.01 | 0.13 | 75816 | - |
| RS4964052 | RS2289709 | 0.616 | 14.81 | 0.036 | 0.5 | 0.71 | 84303 | - |
| RS922270 | RS4964060 | 0.372 | 4.79 | 0.013 | 0.23 | 0.49 | 28416 | 123.49 |
| RS922270 | RS7304939 | 0.421 | 0.8 | 0.0020 | 0.09 | 0.71 | 39394 | - |
| RS922270 | RS12299407 | 0.028 | 0.19 | 0.0010 | -0.01 | 0.09 | 44232 | - |
| RS922270 | RS1037921 | 0.522 | 1.14 | 0.0030 | 0.15 | 0.78 | 48615 | - |
| RS922270 | RS4931075 | 0.751 | 11.63 | 0.028 | 0.6 | 0.85 | 60195 | - |
| RS922270 | RS2289709 | 0.086 | 2.44 | 0.0070 | 0.03 | 0.14 | 68682 | - |
| RS4964060 | RS7304939 | 1.0 | 67.68 | 0.134 | 0.97 | 1.0 | 10978 | 583.01 |
| RS4964060 | RS12299407 | 0.991 | 125.71 | 0.241 | 0.96 | 1.0 | 15816 | - |
| RS4964060 | RS1037921 | 1.0 | 52.64 | 0.103 | 0.96 | 1.0 | 20199 | - |
| RS4964060 | RS4931075 | 0.793 | 151.53 | 0.325 | 0.75 | 0.83 | 31779 | - |
| RS4964060 | RS2289709 | 0.98 | 81.69 | 0.16 | 0.94 | 1.0 | 40266 | - |
| RS7304939 | RS12299407 | 0.722 | 94.14 | 0.286 | 0.66 | 0.78 | 4838 | 1124.63 |
| RS7304939 | RS1037921 | 0.986 | 224.78 | 0.737 | 0.96 | 1.0 | 9221 | - |
| RS7304939 | RS4931075 | 1.0 | 22.8 | 0.042 | 0.92 | 1.0 | 20801 | - |
| RS7304939 | RS2289709 | 0.992 | 277.83 | 0.798 | 0.97 | 1.0 | 29288 | - |
| RS12299407 | RS1037921 | 0.995 | 155.27 | 0.417 | 0.96 | 1.0 | 4383 | 1148.11 |
| RS12299407 | RS4931075 | 0.167 | 4.67 | 0.013 | 0.1 | 0.24 | 15963 | - |
| RS12299407 | RS2289709 | 0.763 | 140.34 | 0.397 | 0.71 | 0.81 | 24450 | - |
| RS1037921 | RS4931075 | 1.0 | 18.56 | 0.033 | 0.9 | 1.0 | 11580 | 906.67 |
| RS1037921 | RS2289709 | 0.981 | 195.18 | 0.593 | 0.95 | 1.0 | 20067 | - |
| RS4931075 | RS2289709 | 1.0 | 29.11 | 0.053 | 0.93 | 1.0 | 8487 | 724.15 |

Figure 9. Haploview LD display of *ARNTL2* SNPs


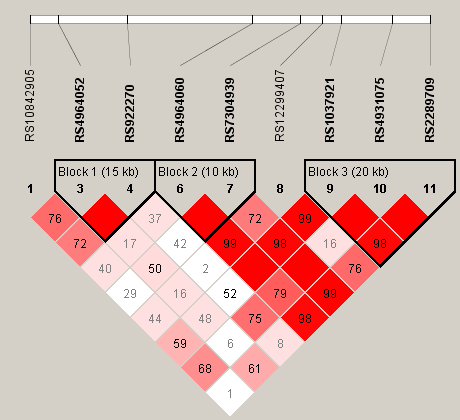


| Table 10: SNPs analyzed in *TIMELESS* gene | | | | | | | | |
| --- | --- | --- | --- | --- | --- | --- | --- | --- |
| L1 | L2 | D' | LOD | r^2 | CIlow | CIhi | Dist | T-int |
| RS2291739 | RS2291738 | 0.995 | 463.77 | 0.758 | 0.98 | 1.0 | 628 | 764.02 |
| RS2291739 | RS7486220 | 0.836 | 278.95 | 0.565 | 0.8 | 0.87 | 22763 | - |
| RS2291739 | RS1082214 | 0.676 | 21.3 | 0.054 | 0.57 | 0.76 | 31837 | - |
| RS2291738 | RS7486220 | 0.839 | 205.88 | 0.435 | 0.8 | 0.87 | 22135 | 520.29 |
| RS2291738 | RS1082214 | 0.626 | 14.16 | 0.035 | 0.5 | 0.73 | 31209 | - |
| RS7486220 | RS1082214 | 0.932 | 14.66 | 0.036 | 0.79 | 0.98 | 9074 | 50.12 |

Figure 10. Haploview LD display of *TIMELESS* SNPs


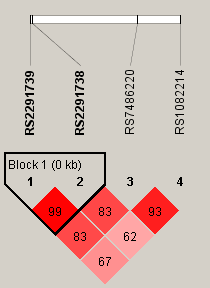


| Table 11: SNPs analyzed in *CRY1* gene | | | | | | | | |
| --- | --- | --- | --- | --- | --- | --- | --- | --- |
| L1 | L2 | D' | LOD | r^2 | CIlow | CIhi | Dist | T-int |
| RS2287162 | RS2287161 | 0.619 | 74.08 | 0.182 | 0.56 | 0.67 | 235 | 134.23 |
| RS2287162 | RS10861683 | 0.629 | 27.78 | 0.07 | 0.54 | 0.7 | 655 | - |
| RS2287162 | RS11113179 | 1.0 | 18.87 | 0.042 | 0.9 | 1.0 | 71880 | - |
| RS2287162 | RS3809237 | 0.248 | 13.5 | 0.038 | 0.19 | 0.31 | 106007 | - |
| RS2287161 | RS10861683 | 0.48 | 32.03 | 0.085 | 0.41 | 0.54 | 420 | 148.87 |
| RS2287161 | RS11113179 | 0.88 | 33.01 | 0.073 | 0.79 | 0.94 | 71645 | - |
| RS2287161 | RS3809237 | 0.294 | 23.68 | 0.066 | 0.24 | 0.35 | 105772 | - |
| RS10861683 | RS11113179 | 1.0 | 18.98 | 0.035 | 0.9 | 1.0 | 71225 | 119.09 |
| RS10861683 | RS3809237 | 0.322 | 11.05 | 0.03 | 0.24 | 0.4 | 105352 | - |
| RS11113179 | RS3809237 | 1.0 | 36.92 | 0.066 | 0.95 | 1.0 | 34127 | 85.15 |

Figure 11. Haploview LD display of *CRY1* SNPs


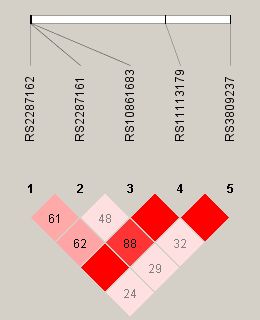


| Table 12: SNPs analyzed in *RORA* gene | | | | | | | | |
| --- | --- | --- | --- | --- | --- | --- | --- | --- |
| L1 | L2 | D' | LOD | r^2 | CIlow | CIhi | Dist | T-int |
| RS2290430 | RS12914584 | 0.0020 | 0.0 | 0.0 | -0.01 | 0.09 | 26132 | 29.9 |
| RS2290430 | RS2028122 | 0.253 | 0.87 | 0.0020 | 0.05 | 0.45 | 49321 | - |
| RS2290430 | RS4775281 | 0.862 | 27.25 | 0.065 | 0.76 | 0.92 | 51817 | - |
| RS2290430 | RS4774370 | 0.084 | 0.77 | 0.0020 | 0.01 | 0.17 | 101477 | - |
| RS2290430 | RS1863270 | 0.31 | 1.01 | 0.0030 | 0.08 | 0.52 | 220305 | - |
| RS2290430 | RS11637301 | 0.667 | 2.74 | 0.0070 | 0.35 | 0.85 | 255944 | - |
| RS2290430 | RS341373 | 0.482 | 2.97 | 0.0090 | 0.26 | 0.66 | 321260 | - |
| RS2290430 | RS8027829 | 0.038 | 0.02 | 0.0 | 0.0 | 0.29 | 381917 | - |
| RS2290430 | RS16943429 | 0.013 | 0.01 | 0.0 | -0.01 | 0.12 | 487299 | - |
| RS12914584 | RS2028122 | 0.972 | 198.79 | 0.418 | 0.94 | 0.99 | 23189 | 324.34 |
| RS12914584 | RS4775281 | 0.942 | 91.15 | 0.196 | 0.9 | 0.97 | 25685 | - |
| RS12914584 | RS4774370 | 0.053 | 0.76 | 0.0020 | 0.0 | 0.11 | 75345 | - |
| RS12914584 | RS1863270 | 0.015 | 0.05 | 0.0 | -0.01 | 0.09 | 194173 | - |
| RS12914584 | RS11637301 | 0.242 | 0.95 | 0.0020 | 0.06 | 0.42 | 229812 | - |
| RS12914584 | RS341373 | 0.04 | 0.06 | 0.0 | -0.01 | 0.18 | 295128 | - |
| RS12914584 | RS8027829 | 0.099 | 1.16 | 0.0040 | 0.02 | 0.18 | 355785 | - |
| RS12914584 | RS16943429 | 0.017 | 0.07 | 0.0 | -0.01 | 0.08 | 461167 | - |
| RS2028122 | RS4775281 | 0.937 | 222.42 | 0.439 | 0.91 | 0.96 | 2496 | 353.64 |
| RS2028122 | RS4774370 | 0.052 | 0.1 | 0.0 | -0.01 | 0.19 | 52156 | - |
| RS2028122 | RS1863270 | 0.035 | 0.08 | 0.0 | -0.01 | 0.15 | 170984 | - |
| RS2028122 | RS11637301 | 0.193 | 1.35 | 0.0030 | 0.06 | 0.32 | 206623 | - |
| RS2028122 | RS341373 | 0.153 | 1.98 | 0.0050 | 0.06 | 0.24 | 271939 | - |
| RS2028122 | RS8027829 | 0.019 | 0.03 | 0.0 | -0.01 | 0.12 | 332596 | - |
| RS2028122 | RS16943429 | 0.118 | 0.63 | 0.0020 | 0.01 | 0.24 | 437978 | - |
| RS4775281 | RS4774370 | 0.051 | 0.19 | 0.0010 | -0.01 | 0.15 | 49660 | 28.89 |
| RS4775281 | RS1863270 | 0.177 | 3.65 | 0.011 | 0.1 | 0.25 | 168488 | - |
| RS4775281 | RS11637301 | 0.375 | 9.33 | 0.025 | 0.27 | 0.47 | 204127 | - |
| RS4775281 | RS341373 | 0.105 | 1.66 | 0.0050 | 0.03 | 0.18 | 269443 | - |
| RS4775281 | RS8027829 | 0.012 | 0.03 | 0.0 | -0.01 | 0.08 | 330100 | - |
| RS4775281 | RS16943429 | 0.019 | 0.03 | 0.0 | -0.01 | 0.12 | 435482 | - |
| RS4774370 | RS1863270 | 0.168 | 6.17 | 0.018 | 0.11 | 0.23 | 118828 | 44.18 |
| RS4774370 | RS11637301 | 0.094 | 2.65 | 0.0070 | 0.04 | 0.15 | 154467 | - |
| RS4774370 | RS341373 | 0.076 | 0.25 | 0.0010 | 0.0 | 0.2 | 219783 | - |
| RS4774370 | RS8027829 | 0.214 | 2.22 | 0.0060 | 0.09 | 0.33 | 280440 | - |
| RS4774370 | RS16943429 | 0.126 | 5.08 | 0.014 | 0.07 | 0.18 | 385822 | - |
| RS4774370 | RS1568717 | 0.062 | 1.08 | 0.0030 | 0.01 | 0.12 | 469016 | - |
| RS1863270 | RS11637301 | 0.213 | 1.26 | 0.0040 | 0.06 | 0.36 | 35639 | 34.97 |
| RS1863270 | RS341373 | 0.0070 | 0.0 | 0.0 | -0.01 | 0.12 | 100955 | - |
| RS1863270 | RS8027829 | 0.214 | 3.4 | 0.01 | 0.12 | 0.31 | 161612 | - |
| RS1863270 | RS16943429 | 0.074 | 1.33 | 0.0040 | 0.02 | 0.13 | 266994 | - |
| RS1863270 | RS1568717 | 0.101 | 0.42 | 0.0010 | 0.01 | 0.23 | 350188 | - |
| RS1863270 | RS893287 | 0.082 | 1.62 | 0.0050 | 0.02 | 0.14 | 381973 | - |
| RS1863270 | RS4774388 | 0.053 | 0.06 | 0.0 | 0.0 | 0.23 | 454739 | - |
| RS1863270 | RS1816624 | 0.055 | 0.1 | 0.0 | -0.01 | 0.21 | 457063 | - |
| RS11637301 | RS341373 | 0.954 | 42.24 | 0.089 | 0.89 | 0.99 | 65316 | 64.64 |
| RS11637301 | RS8027829 | 0.241 | 2.28 | 0.0070 | 0.11 | 0.36 | 125973 | - |
| RS11637301 | RS16943429 | 0.035 | 0.35 | 0.0010 | -0.01 | 0.09 | 231355 | - |
| RS11637301 | RS1568717 | 0.0060 | 0.01 | 0.0 | -0.01 | 0.07 | 314549 | - |
| RS11637301 | RS893287 | 0.0040 | 0.0 | 0.0 | -0.01 | 0.09 | 346334 | - |
| RS11637301 | RS4774388 | 0.22 | 0.52 | 0.0020 | 0.03 | 0.44 | 419100 | - |
| RS11637301 | RS1816624 | 0.0010 | 0.0 | 0.0 | -0.01 | 0.06 | 421424 | - |
| RS11637301 | RS6494251 | 0.121 | 4.38 | 0.012 | 0.07 | 0.18 | 466669 | - |
| RS11637301 | RS10438343 | 0.093 | 2.63 | 0.0070 | 0.04 | 0.15 | 475682 | - |
| RS341373 | RS8027829 | 0.274 | 21.89 | 0.064 | 0.22 | 0.32 | 60657 | 50.25 |
| RS341373 | RS16943429 | 0.217 | 2.06 | 0.0060 | 0.09 | 0.34 | 166039 | - |
| RS341373 | RS1568717 | 0.113 | 2.69 | 0.0080 | 0.05 | 0.17 | 249233 | - |
| RS341373 | RS893287 | 0.222 | 4.53 | 0.013 | 0.13 | 0.31 | 281018 | - |
| RS341373 | RS4774388 | 0.077 | 0.65 | 0.0020 | 0.0 | 0.16 | 353784 | - |
| RS341373 | RS1816624 | 0.056 | 0.5 | 0.0010 | 0.0 | 0.13 | 356108 | - |
| RS341373 | RS6494251 | 0.053 | 0.12 | 0.0 | -0.01 | 0.18 | 401353 | - |
| RS341373 | RS10438343 | 0.016 | 0.01 | 0.0 | -0.01 | 0.16 | 410366 | - |
| RS8027829 | RS16943429 | 0.026 | 0.03 | 0.0 | -0.01 | 0.15 | 105382 | 23.33 |
| RS8027829 | RS1568717 | 0.075 | 0.32 | 0.0010 | 0.0 | 0.19 | 188576 | - |
| RS8027829 | RS893287 | 0.066 | 0.47 | 0.0010 | 0.0 | 0.15 | 220361 | - |
| RS8027829 | RS4774388 | 0.096 | 0.86 | 0.0030 | 0.01 | 0.19 | 293127 | - |
| RS8027829 | RS1816624 | 0.07 | 0.65 | 0.0020 | 0.0 | 0.15 | 295451 | - |
| RS8027829 | RS6494251 | 0.037 | 0.21 | 0.0010 | -0.01 | 0.11 | 340696 | - |
| RS8027829 | RS10438343 | 0.036 | 0.19 | 0.0010 | -0.01 | 0.11 | 349709 | - |
| RS16943429 | RS1568717 | 0.089 | 2.45 | 0.0070 | 0.04 | 0.14 | 83194 | 27.83 |
| RS16943429 | RS893287 | 0.075 | 0.29 | 0.0010 | 0.0 | 0.19 | 114979 | - |
| RS16943429 | RS4774388 | 0.322 | 1.73 | 0.0040 | 0.13 | 0.49 | 187745 | - |
| RS16943429 | RS1816624 | 0.177 | 0.72 | 0.0020 | 0.03 | 0.34 | 190069 | - |
| RS16943429 | RS6494251 | 0.116 | 4.53 | 0.013 | 0.06 | 0.17 | 235314 | - |
| RS16943429 | RS10438343 | 0.105 | 3.59 | 0.01 | 0.05 | 0.16 | 244327 | - |
| RS1568717 | RS893287 | 0.921 | 65.77 | 0.139 | 0.86 | 0.96 | 31785 | 111.18 |
| RS1568717 | RS4774388 | 0.12 | 0.24 | 0.0010 | 0.01 | 0.31 | 104551 | - |
| RS1568717 | RS1816624 | 0.025 | 0.02 | 0.0 | -0.01 | 0.2 | 106875 | - |
| RS1568717 | RS6494251 | 0.174 | 9.25 | 0.026 | 0.12 | 0.23 | 152120 | - |
| RS1568717 | RS10438343 | 0.175 | 9.32 | 0.026 | 0.12 | 0.23 | 161133 | - |
| RS893287 | RS4774388 | 0.113 | 0.42 | 0.0010 | 0.01 | 0.25 | 72766 | 38.66 |
| RS893287 | RS1816624 | 0.074 | 0.25 | 0.0010 | 0.0 | 0.2 | 75090 | - |
| RS893287 | RS6494251 | 0.121 | 2.62 | 0.0070 | 0.05 | 0.19 | 120335 | - |
| RS893287 | RS10438343 | 0.127 | 2.78 | 0.0070 | 0.06 | 0.2 | 129348 | - |
| RS4774388 | RS1816624 | 0.982 | 286.45 | 0.68 | 0.96 | 1.0 | 2324 | 351.4 |
| RS4774388 | RS6494251 | 0.872 | 13.66 | 0.03 | 0.72 | 0.95 | 47569 | - |
| RS4774388 | RS10438343 | 0.966 | 17.16 | 0.036 | 0.84 | 1.0 | 56582 | - |
| RS1816624 | RS6494251 | 0.894 | 20.66 | 0.045 | 0.78 | 0.95 | 45245 | 107.62 |
| RS1816624 | RS10438343 | 0.955 | 24.05 | 0.051 | 0.86 | 0.99 | 54258 | - |
| RS6494251 | RS10438343 | 0.943 | 403.22 | 0.849 | 0.92 | 0.96 | 9013 | 456.53 |

Figure 12.Haploview LD display of *RORA* SNPs


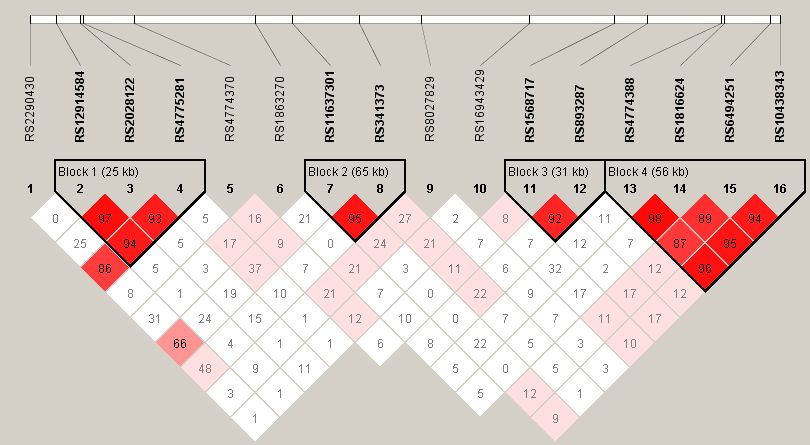


| Table 13: SNPs analyzed in *TIPIN* gene | | | | | | | | |
| --- | --- | --- | --- | --- | --- | --- | --- | --- |
| L1 | L2 | D' | LOD | r^2 | CIlow | CIhi | Dist | T-int |
| RS3759785 | RS3759786 | 0.796 | 1.19 | 0.0040 | 0.21 | 0.98 | 3933 | 115.19 |
| RS3759785 | RS2063690 | 1.0 | 105.01 | 0.397 | 0.97 | 1.0 | 16262 | - |
| RS3759785 | RS8031897 | 1.0 | 8.99 | 0.013 | 0.81 | 1.0 | 20621 | - |
| RS3759786 | RS2063690 | 1.0 | 4.8 | 0.016 | 0.6 | 1.0 | 12329 | 154.14 |
| RS3759786 | RS8031897 | 1.0 | 35.34 | 0.066 | 0.94 | 1.0 | 16688 | - |
| RS2063690 | RS8031897 | 1.0 | 20.77 | 0.034 | 0.91 | 1.0 | 4359 | 65.1 |

Figure 13.Haploview LD display of *TIPIN*  SNPs


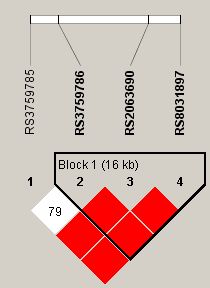


| Table 14: SNPs analyzed in *NR1D1* gene | | | | | | | | |
| --- | --- | --- | --- | --- | --- | --- | --- | --- |
| L1 | L2 | D' | LOD | r^2 | CIlow | CIhi | Dist | T-int |
| RS2314339 | RS2071427 | 0.843 | 135.12 | 0.319 | 0.8 | 0.88 | 1280 | 424.16 |
| RS2314339 | RS2269457 | 0.887 | 178.82 | 0.41 | 0.85 | 0.92 | 1477 | - |
| RS2314339 | RS2071570 | 0.735 | 110.22 | 0.283 | 0.68 | 0.78 | 3878 | - |
| RS2071427 | RS2269457 | 0.67 | 168.5 | 0.39 | 0.63 | 0.71 | 197 | 522.09 |
| RS2071427 | RS2071570 | 0.452 | 64.55 | 0.175 | 0.41 | 0.5 | 2598 | - |
| RS2269457 | RS2071570 | 0.694 | 211.16 | 0.477 | 0.66 | 0.73 | 2401 | 385.93 |

Figure 14.Haploview LD display of *NR1D1*  SNPs


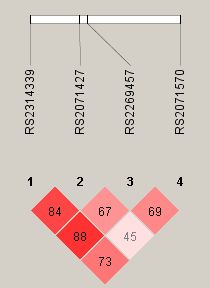


| Table 15: SNPs analyzed in *PER1* gene | | | | | | | | |
| --- | --- | --- | --- | --- | --- | --- | --- | --- |
| L1 | L2 | D' | LOD | r^2 | CIlow | CIhi | Dist | T-int |
| RS2289591 | RS2253820 | 1.0 | 19.61 | 0.035 | 0.9 | 1.0 | 159 | 159.26 |
| RS2289591 | RS3027188 | 1.0 | 15.31 | 0.028 | 0.88 | 1.0 | 975 | - |
| RS2289591 | RS885747 | 0.986 | 116.83 | 0.221 | 0.95 | 1.0 | 2727 | - |
| RS2289591 | RS2518023 | 0.927 | 7.51 | 0.015 | 0.7 | 0.98 | 8596 | - |
| RS2253820 | RS3027188 | 0.982 | 272.8 | 0.746 | 0.96 | 1.0 | 816 | 583.22 |
| RS2253820 | RS885747 | 0.479 | 12.37 | 0.032 | 0.37 | 0.58 | 2568 | - |
| RS2253820 | RS2518023 | 0.989 | 158.4 | 0.472 | 0.96 | 1.0 | 8437 | - |
| RS3027188 | RS885747 | 0.959 | 48.19 | 0.099 | 0.9 | 0.99 | 1752 | 532.27 |
| RS3027188 | RS2518023 | 0.989 | 188.97 | 0.614 | 0.96 | 1.0 | 7621 | - |
| RS885747 | RS2518023 | 0.983 | 32.84 | 0.064 | 0.91 | 1.0 | 5869 | 387.72 |

Figure 15.Haploview LD display of *PER1*  SNPs


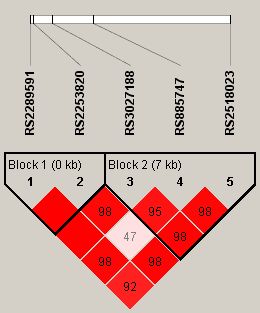


| Table 16: SNPs analyzed in *CSNK1E* gene | | | | | | | | |
| --- | --- | --- | --- | --- | --- | --- | --- | --- |
| L1 | L2 | D' | LOD | r^2 | CIlow | CIhi | Dist | T-int |
| RS135745 | RS2075984 | 0.02 | 0.12 | 0.0 | -0.01 | 0.07 | 7252 | 27.69 |
| RS135745 | RS5750581 | 0.716 | 25.55 | 0.062 | 0.62 | 0.79 | 11769 | - |
| RS135745 | RS7289981 | 0.207 | 2.02 | 0.0050 | 0.09 | 0.32 | 36325 | - |
| RS2075984 | RS5750581 | 1.0 | 68.04 | 0.134 | 0.97 | 1.0 | 4517 | 99.98 |
| RS2075984 | RS7289981 | 0.289 | 4.37 | 0.012 | 0.17 | 0.4 | 29073 | - |
| RS5750581 | RS7289981 | 0.057 | 0.02 | 0.0 | 0.0 | 0.4 | 24556 | 6.41 |

Figure 16.Haploview LD display of *CSNK1E*  SNPs


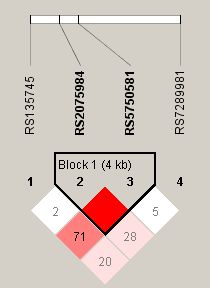

Supplement: Table S4 — Linkage Disequilibrium (LD) patterns for all genotyped variations within each of the studied circadian genes in the Health 2000 dataset. (1.00 MB DOC) [file pone.0009259.s006.doc]
